# Antibody Evasion by a Gammaherpesvirus O-Glycan Shield

**DOI:** 10.1371/journal.ppat.1002387

**Published:** 2011-11-17

**Authors:** Bénédicte Machiels, Céline Lété, Antoine Guillaume, Jan Mast, Philip G. Stevenson, Alain Vanderplasschen, Laurent Gillet

**Affiliations:** 1 Immunology-Vaccinology (B43b), Department of Infectious and Parasitic Diseases (B43b), Faculty of Veterinary Medicine, University of Liège, Liège, Belgium; 2 Department Biocontrole, Research Unit Electron Microscopy, Veterinary and Agrochemical Research Centre, CODA-CERVA, Groeselenberg, Ukkel, Belgium; 3 Division of Virology, Department of Pathology, University of Cambridge, Cambridge, United Kingdom; University of North Carolina at Chapel Hill, United States of America

## Abstract

All gammaherpesviruses encode a major glycoprotein homologous to the Epstein-Barr virus gp350. These glycoproteins are often involved in cell binding, and some provide neutralization targets. However, the capacity of gammaherpesviruses for long-term transmission from immune hosts implies that *in vivo* neutralization is incomplete. In this study, we used Bovine Herpesvirus 4 (BoHV-4) to determine how its gp350 homolog - gp180 - contributes to virus replication and neutralization. A lack of gp180 had no impact on the establishment and maintenance of BoHV-4 latency, but markedly sensitized virions to neutralization by immune sera. Antibody had greater access to gB, gH and gL on gp180-deficient virions, including neutralization epitopes. Gp180 appears to be highly O-glycosylated, and removing O-linked glycans from virions also sensitized them to neutralization. It therefore appeared that gp180 provides part of a glycan shield for otherwise vulnerable viral epitopes. Interestingly, this O-glycan shield could be exploited for neutralization by lectins and carbohydrate-specific antibody. The conservation of O-glycosylation sites in all gp350 homologs suggests that this is a general evasion mechanism that may also provide a therapeutic target.

## Author Summary

Herpesvirus transmission between immune hosts implies some kind of antibody evasion. However, the underlying molecular mechanisms remain largely unknown. All gammaherpesviruses encode a major glycoprotein homologous to the Epstein-Barr virus (EBV) gp350. Gp350 binds EBV to B cells and provides a neutralization target. However, despite its immunogenicity, EBV carriers remain infectious. Here we show that the gp350 homolog of the related Bovine Herpesvirus 4 (BoHV-4), gp180, and its O-glycans, shield some otherwise vulnerable viral epitopes. Extensive O-glycosylation is common to all gammaherpesvirus gp350 homologs, suggesting that this evasion mechanism is also widespread.

## Introduction

Epstein-Barr virus (EBV) and Kaposi’s Sarcoma Associated Herpesvirus (KSHV) are DNA tumor viruses that provide risk factors for Burkitt's lymphoma, Hodgkin's lymphoma, nasopharyngeal carcinoma, Kaposi's Sarcoma and post-transplant lymphoproliferative disease [1]–[2]. EBV infection has also been associated with multiple sclerosis [3]–[4]. Healthy carriers consistently shed virus in saliva [5] that infects naïve individuals [6]–[7] despite being exposed to virus-specific antibody [8]–[9]. This lack of neutralization contrasts completely with non-persistent mucosal infections such as that of poliovirus [10]–[11], and implies that gammaherpesviruses have evolved specific antibody evasion mechanisms.

Neutralizing antibodies generally target epitopes involved in virion binding or membrane fusion [12]. Targeting of the gB/gH/gL [13]–[16] fusion machinery [17]–[18] seems to be limited by a paucity of good targets [19] and poor immunogenicity [20]. Therefore most studies have looked at binding. The EBV gp350 is an abundant component of the virion envelope that binds to CD21 on B cells [21]–[22] and is a target for antibodies that neutralize B cell infection [23]. However, while EBV lacking gp350 is poorly infectious for B cells [24]–[25], it infects CD21-negative epithelial cells better than the wild-type [25], and these may provide a primary target for virions entering naive hosts. Epithelial infection can even be enhanced by gp350-specific antibodies [26]. Therefore the relationship between EBV transmission, gp350, and gp350-specific antibodies needs further exploration, particularly as gp350 is a candidate EBV vaccine [27]–[28].

Our understanding of EBV and KSHV is limited by their narrow species tropisms. Related animal viruses are therefore an important source of information. Two of the best established experimental models are provided by Murid herpesvirus 4 (MuHV-4) [29] and Bovine herpesvirus 4 (BoHV-4) [30]–[31]. Their homologs of gp350 are gp150 in MuHV-4 [32], encoded by M7, and gp180 in BoHV-4 [33], encoded by Bo10. While these proteins are diverse in sequence, they seem to be related in function, being involved in both binding to a cellular receptor and in blocking the infection of cells that do not express this receptor [25], [32]–[33]. It has been proposed that the receptor interaction displaces each homolog to reveal other glycoproteins involved in entry. Thus, a non-essential glycoprotein [24], [32]–[33] could hide from neutralization some critical epitopes on cell-free virions.

To date, the *in vivo* function of gp350 homologs has only been investigated with MuHV-4. Surprisingly, gp150-deficient viruses showed only a transient lag in lytic replication *in vivo* and established normal levels of latency [32]. Gp150 is the most immunogenic MuHV-4 glycoprotein and anti-gp150 antibodies play a predominant role in driving Fc receptor-dependent infection [20]. While gp150 does not have an obvious direct role in cell-binding, BoHV-4 lacking gp180 displays a binding deficit [33]. Therefore this protein may be more closely analogous to gp350 and the KSHV K8.1 than is gp150. Here we investigated the consequences of gp180 deletion for BoHV-4 replication *in vivo* and neutralization. An important gp180 function seemed to be to block the binding to virions of antibodies that would otherwise neutralize.

## Results

### Generation of a Bo10 nonsense BoHV-4 mutant

**Figure 1:**
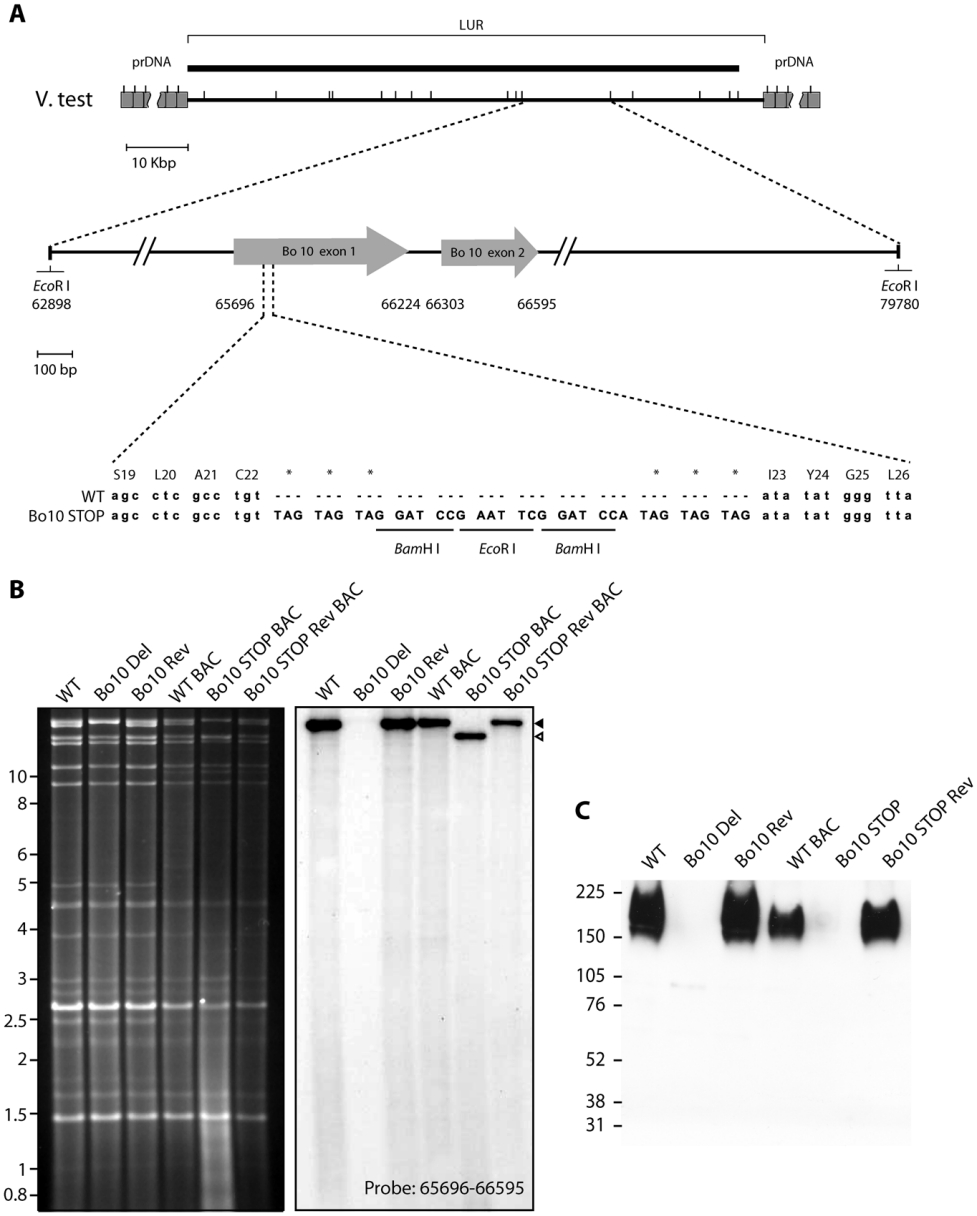
Generation of a Bo10 STOP BoHV-4 mutant. **A**. Schematic representation of the strategy followed to produce the recombinant BoHV-4 strains. The Bo10 STOP BoHV-4 mutant was derived from a cloned BoHV-4 BAC by *gal*K-counterselection method. The Bo10 coding sequence was disrupted by inserting stop codons near the end of the coding sequence for its predicted signal peptide (Bo10 STOP). The mutation incorporated two *Bam*HI and one *Eco*RI restriction sites. **B**. Verification of the molecular structure. Viral DNA was digested with *Eco*RI, resolved by agarose gel electrophoresis, and hybridized with a ^32^P-labeled probe, corresponding to nucleotides 65,696–66,595 of the BoHV-4 V.test strain genome. Black triangle shows the restriction fragment that contains the WT Bo10 gene. The Bo10 Del fragment is higher due to eGFP insertion [33], however, it is much less visible due to deletion of most of the ORF. The 16,882-bp wild-type (WT) band becomes 14,033-bp (open triangle) for the Bo10 STOP mutant. The 2,885 bp band is not visible because it only hybridizes with a few nucleotides of the probe. In WT BAC, Bo10 STOP BAC and Bo10 STOP BAC Rev strains, the 4,870 bp band becomes 9,964 bp and 3,416 bp due to BAC cassette insertion as described previously [30]. Marker sizes in Kbp are indicated on the left. **C**. Detection of the Bo10 encoded gp180 protein by the anti-Bo10-c15 serum. Purified virions (5*10^5^ virions per lane) were subjected to western blotting with anti-Bo10-c15 serum as described in the Material and Methods. The position of a molecular mass (MM) standard (in kDa) is shown on the left.

We previously described a BoHV-4 strain in which the entire Bo10 ORF was replaced by an eGFP expression cassette [33]. Since expression cassettes can cause *in vivo* attenuation, we also generated a second Bo10 mutant virus, in which stop codons terminated Bo10 translation 7 amino acids before the end of its predicted signal sequence without any associated deletion (Figure 1A). A revertant strain, called Bo10 STOP Rev, was finally constructed to validate the Bo10 STOP mutant. The predicted molecular structures of the recombinant strains were confirmed by *Eco*RI restriction mapping and Southern blotting (Figure 1B), and further by DNA sequencing. Immunoblotting with an anti-Bo10-c15 rabbit polyserum [33] established that the Bo10 mutant virions lacked gp180 (Figure 1C).

### Dissemination of Bo10- mutants *in vivo*

**Figure 2:**
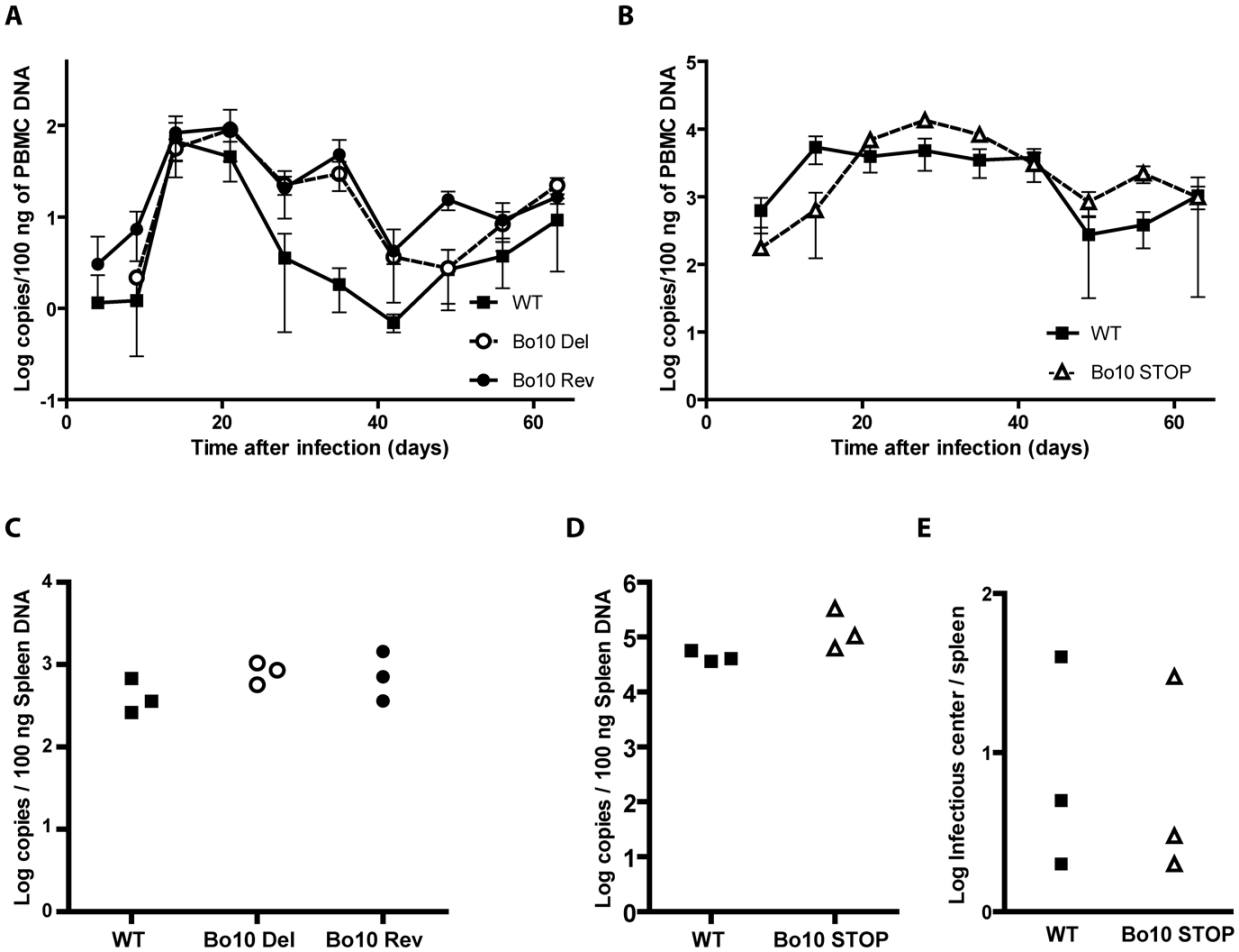
*In vivo* persistence of Bo10- mutants. Groups consisting of 3 rabbits were mock-infected or infected with 10^8^ PFU of BoHV-4 WT V.test, Bo10 Del and Bo10 Rev strains (A and C) or infected with 10^7^ PFU of BoHV-4 WT V.test and Bo10 STOP excised strains (B, D and E). **A–D**. Real-Time PCR relative quantification of BoHV-4 genomes. DNA was extracted from the PBMC (A and B) at the different times post-inoculation and from the spleen (C and D) 64 days post-inoculation. Data are expressed as the number of BoHV-4 ORF8 gene copies per 100 ng of total DNA. In A and B, the data presented are the average ± SEMs and were analyzed by 2ways ANOVA and Bonferroni posttests. In C and D, each point shows the genome copies for one rabbit. The data were analyzed by 1way ANOVA and Bonferroni posttests or Student t-test. No significative difference was observed. **E**. Spleens from the different rabbits were analyzed individually for reactivable BoHV-4 by infectious-center assay. Each point shows the infectious centers for one rabbit. The data were analyzed by Student t-test. No significative difference was observed.

To investigate the importance of Bo10 *in vivo*, we infected rabbits with the different viral strains as described in the Material and Methods. No rabbit showed clinical disease or noticeable pathology at necropsy 64 days post-inoculation. Host colonization was assayed by quantitative PCR of DNA from peripheral blood mononuclear cells (PBMC) over time (Figure 2A and B) and from the spleens at 64 days post-inoculation (Figure 2C and D). The Bo10 mutants showed no deficit. We further performed infectious center assays on spleen cells from the WT and Bo10 STOP infected rabbits. Viral plaques were observed in all samples (Figure 2E). No preformed infectious virus was detected in the equivalent freeze-thawed samples (data not shown), so this was latent infection. Thus, we detected no difference in acute replication, latency establishment or reactivation of Bo10-deficient mutants compared to WT or revertant strains.

### Increased susceptibility of Bo10- BoHV-4 to serum neutralization

**Figure 3:**
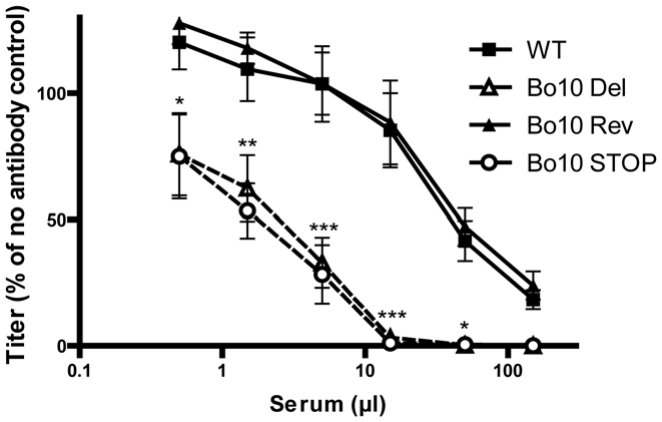
Neutralization of the BoHV-4 Bo10 mutants. BoHV-4 WT V.test, Bo10 Del, Bo10 Rev and Bo10 STOP virions were incubated with sera of 4 different rabbits infected with BoHV-4 V.test strain. After incubation (2 h, 37°C) the viruses were plaque assayed for infectivity on MDBK cells. BoHV-4 titers are expressed relative to virus without antibody. The data presented are the average ± SEMs for 4 measurements and were analyzed by 2way ANOVA and Bonferroni posttests, * p<0.05, ** p<0.01, *** p<0.001.

While pathogenesis assays are a useful measure of viral fitness, they do not measure all viral functions. In particular, virion susceptibility to neutralization [33] might not be measured because intra-host dissemination depends mainly on cell/cell virus spread and latency-associated cell proliferation. We therefore further compared the sensitivity of BoHV-4 WT, Bo10 Del, Bo10 Rev and Bo10 STOP strains to neutralization by sera of rabbits infected with the BoHV-4 V.test strain (Figure 3). WT and Bo10 Rev virions were poorly neutralized. Bo10 Del and Bo10 STOP virions were neutralized much better. In particular, complete neutralization was now possible. Neutralization experiments with eGFP expressing viruses on different cell types confirmed this result, with gp180-deficient virions showing increased sensitivity to neutralization by anti-BoHV-4 serum compared to WT virions (Figure S1). Thus gp180 seemed to limit virion neutralization.

### Bo10 sequence variation

**Figure 4:**
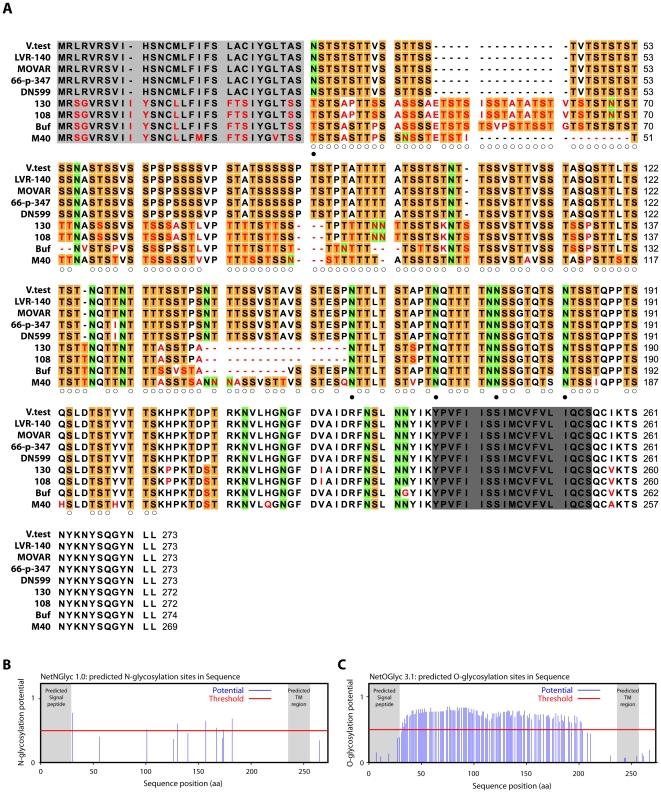
Sequence analysis of the Bo10 encoded proteins of nine BoHV-4 strains. **A**. BoHV-4 Bo10 encoded proteins alignment. Predicted Bo10 transmembrane protein encoded by nine different BoHV-4 strains were aligned (ClustalX; [88]). Predicted peptide signals [89] and transmembrane regions were highlighted in pale and dark grey respectively. Non-conserved residues were printed in red. Serine (S) and threonine (T) residues of the N-terminal ectodomain were highlighted in orange, asparagine (N) residues of the N-terminal ectodomain were highlighted in green. Open and filled circles indicate potential O- and N-glycosylation sites respectively (using NetNglyc 1.0 and NetOglyc 3.1 algorithms [37]) that are predicted for each of these residues in all the different strains that display such residue at that position. **B**. Prediction of N-glycosylation sites for the complete BoHV-4 V.test gp180 protein sequence using the NetNglyc 1.0 algorithm. The shaded regions indicate the signal peptide and transmembrane region. The red line indicates significative threshold. **C**. Prediction of O-glycosylation sites for the complete BoHV-4 V.test gp180 protein sequence using the NetOglyc 3.1 algorithm. The shaded regions indicate the signal peptide and transmembrane region. The red line indicates significative threshold.

In order to understand how gp180 might protect virion against neutralization, we compared the Bo10 genes of different BoHV-4 isolates [34]. All showed consensus splice donor and acceptor sites that are used in the BoHV-4 V.test strain to generate gp180 [33]. Nucleotide sequences comparison across the entire open reading frames revealed up to 15% inter-strain divergence (Table S1). Amino acid divergence between American-European and African strains reached 39%, mostly in the N-terminal half of the protein ectodomain (Figure 4A). By comparison, gB, gH and gL differ by <2% between KSHV strains [35]; gB differs by <2% between OHV-2 strains [36]; and ORF71 differs by only 5% between BoHV-4 strains. All gp180s were extremely rich in serine and threonine residues, which accounted for 54.3 +/− 0.6% of each mature ectodomain (Figure 4A). Asparagine residues accounted for a further 8.0 +/− 0.6%. Therefore, a conserved feature seemed to be extensive O- and N-linked glycosylation (Figure 4A–C) [37].

### Gp180 is O-glycosylated

**Figure 5:**
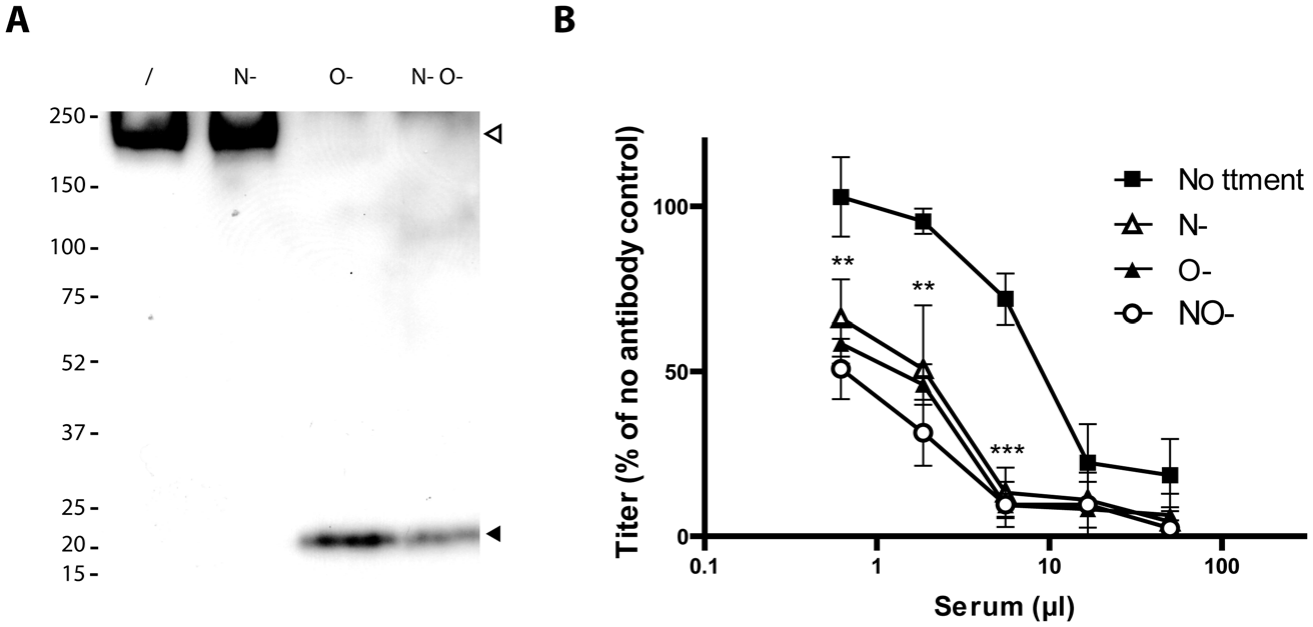
Glycosylation of BoHV-4 gp180 and importance of BoHV-4 glycans in neutralization evasion. **A**. Purified BoHV-4 WT V.test strain virions were deglycosylated after denaturation. /, no enzyme; N-, protein N-glycanase; O-, sialidase A + β1-4 Galactosidase + O-glycanase; N O-, protein N-glycanase + sialidase A + β1-4 Galactosidase + O-glycanase. Each was then immunoblotted for gp180 with anti-Bo10-c15 serum as described in the Material and Methods. The position of a MM standard (in kDa) is shown on the left. **B**. Intact MDBK cell-derived BoHV-4 V.test strain WT virions were deglycosylated without denaturation. /, no enzyme, N-, protein N-glycanase, O-, sialidase A + O-glycanase, N-O- protein N-glycanase + sialidase A + O-glycanase. Each was then tested for neutralization by serum of rabbits immunized by the BoHV-4 V.test strain. After incubation (2 h, 37°C) the viruses were plaque assayed for infectivity on MDBK cells. BoHV-4 titers are expressed relative to virus without antibody. The data presented are the average ± SEMs for 4 measurements and were analyzed by 2way ANOVA and Bonferroni posttests, ** p<0.01, *** p<0.001. Statistical significance was only shown for O- treatment. Equivalent data were obtained in two further experiments.

The Bo10 gene product of BoHV-4 V.test has 122 and 7 potential O- and N-glycosylation sites respectively (Figure 4) [37]. This protein has a predicted molecular mass (MM) of 25 kDa but an apparent MM of 180 kDa [33]. To establish the contribution of glycans to the apparent MM, we digested virions lysates with glycanases. We removed high mannose, hybrid and complex N-glycans [38] with PNGase F. We removed O-glycans successively with sialidase A, β1-4 Galactosidase and O-glycanase. While PNGase F did not affect the apparent MM of gp180 (Figure 5A), removing O-glycans reduced it to approximately 20 kDa, consistent with its predicted unglycosylated MM. Therefore, gp180 was extensively O-glycosylated and O-glycans appear to account for most of its mass.

### Glycans protect BoHV-4 against neutralization

To test whether O-glycans protect BoHV-4 against neutralization, we removed N- and/or O-glycans from intact virions before testing their susceptibility to neutralization. As removing glycans itself could affect viral titers (Figure S2), we expressed the results as a percentage of the number of plaques for each treatment without neutralization. As observed for other viral species [39]–[41], removing N-glycans increased virion susceptibility to neutralization by immune serum (Figure 5B). Removing O-glycans had a similar effect (Figure 5B). Therefore BoHV-4 uses both N- and O-linked glycans to limit its neutralization. Interestingly, gp180 bears most of the predicted BoHV-4 envelope O-glycans (122/155) [37]. These results suggest therefore that gp180 O-glycans provide part of a glycan shield for otherwise vulnerable viral epitopes.

### Altered antigenicity of BoHV-4 lacking gp180

Our subsequent analysis focused on the identification of the neutralization epitopes hidden by gp180. The MuHV-4 gp150 seems to form a multiprotein entry complex with gB, gH and gL [42]. We therefore focused on antibodies raised against the BoHV-4 gB, gH and gL. Monoclonal antibodies (mAbs) were screened for gB, gH, gL or gH/gL specificity as described in the Material and Methods. Mabs 16, 29 and 33 recognize gL, gB and the heterodimer gH/gL, respectively (Figure S3). Mab 35 recognizes gB as previously stated [43].

**Figure 6:**
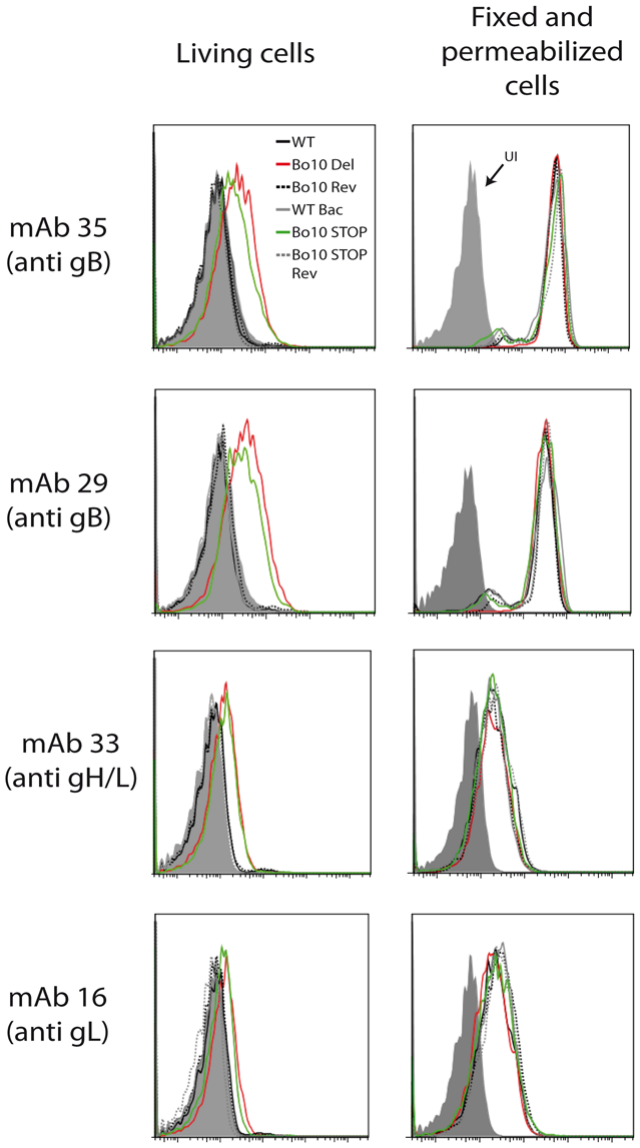
Antigenicity of infected cells. MDBK cells were infected (2 PFU/cell, 36 h) with WT (solid black lines), Bo10 Del (red lines), Bo10 Rev (dotted black lines), WT BAC (grey lines), Bo10 STOP (green lines) or Bo10 STOP Rev (dotted grey lines) of BoHV-4 V.test, and then analyzed by flow cytometry. The filled histogram shows uninfected cells. MAb 29 and 35 recognize gB, mAb 16 recognizes gL, and mAb 33 recognizes gH/L. Fixed (PFA 4%, 4°C for 30 min) and permeabilized (saponin 0.1%) cells were used as control of protein expression.

As with MuHV-4 [32], infected cell surfaces provide a means of probing antigenic differences between BoHV-4 glycoprotein mutants. We compared cells infected by WT, Bo10 Del, Bo10 Rev, WT BAC, Bo10 STOP and Bo10 STOP Rev BoHV-4 viruses. MAbs 29 and 35 (recognizing gB), mAb 16 (recognizing gL) and mAb 33 (recognizing the gH/gL complex) all stained cells infected with the Bo10 Del and Bo10 STOP strains better than they stained those infected with wild-type or revertant viruses (Figure 6). This result was not due to differences in protein expression, as permeabilized cells gave similar staining with each virus (Figure 6).

**Figure 7:**
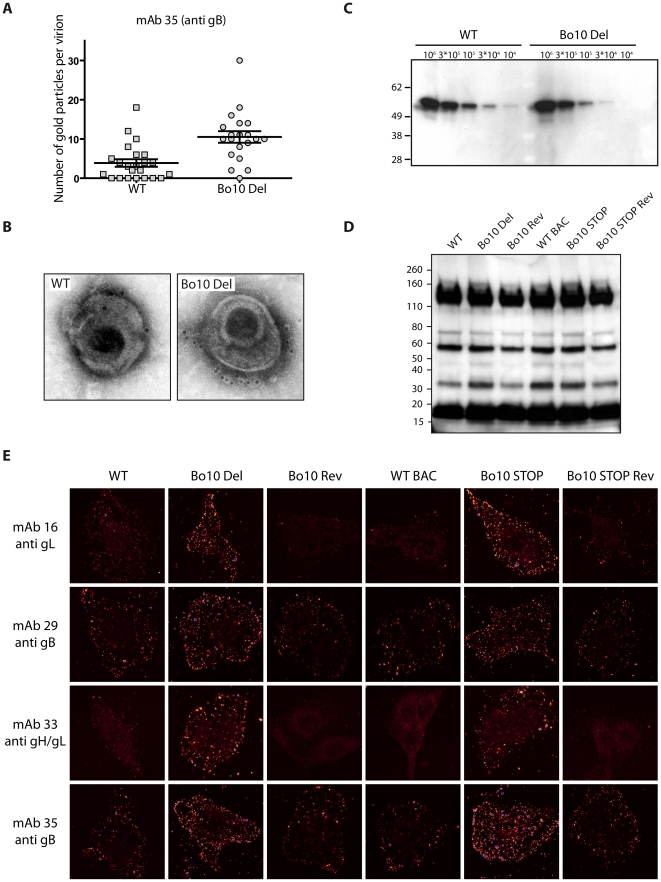
Antigenicity of purified virions. **A**. **and** **B**. Immuno-electron microscopy. Purified BoHV-4 WT V.test or Bo10 Del virions were processed for immune-electron microscopy as described in Material and Methods. These samples were stained for gB with mAb 35 followed by secondary goat anti-mouse IgG-10 nm gold labeled. Pictures of individual virions were then taken and gold particles were counted on each virion. The data presented are the average ± SEMs for 20 different virions and were analyzed by Student’s *t*-test, *** p<0.001 (A). Pictures of representative virions are showed (B). **C**. Purified BoHV-4 WT V.test and Bo10 Del virus stocks were compared for gB content per PFU by immunoblotting for gB (with mAb 35) as described in the Material and Methods. The position of a MM standard (in kDa) is shown on the left. **D**. Purified BoHV-4 WT V.test, Bo10 Del, Bo10 Rev, WT BAC, Bo10 STOP and Bo10 STOP Rev virions (10^5^ PFU per lane) were compared for particles content per PFU by immunoblotting with anti BoHV-4 V.test polyserum as described in the Material and Methods. The position of a MM standard (in kDa) is shown on the left. **E**. Antigenicity of bound purified virions. MDBK cells were exposed to BoHV-4 V.test WT, Bo10 Del, Bo10 Rev, WT BAC, Bo10 STOP and Bo10 STOP Rev virions (20 PFU/cell, 2 h, 4°C), then directly fixed with acetone 95%/water 5%. The cells were then stained for gB with mAb 29 and 35, for gL with mAb 16 and for gH/gL with mAb 33. The pictures were taken with a confocal laser microscope. Images are shown with a pseudo-color glow scale (White, highest to black, lowest intensity, blue represents overexposed pixels).

We then analyzed WT and Bo10 Del virions by immunogold labeling with mAb 35 raised against gB. While binding of gold particles was observed with both strains (Figure 7A), there were statistically more particles on Bo10 Del virions than on WT virions (p<0.001). Representative particules are shown in Figures 7B and S4. This difference did not reflect a greater gB content of Bo10 del virions, since immunoblotting on the same viral preparations with the same antibody showed equivalent signals between the mutant and the WT (Figure 7C). The different stocks displayed also similar particle/PFU ratios as shown in Figure 7D. Finally, increased accessibility of some epitopes on Bo10 mutant virions was confirmed by immunofluorescence (Figure 7E) of virions bound to cell surfaces. The cells were scanned by confocal microscopy with settings unchanged between different viruses stained with the same antibody. Glow pseudo-color analysis established that the staining was stronger when Bo10 was deleted. The difference was particularly evident for mAbs 16 (anti-gL) and 33 (anti gH/L) (Figure 7E). Together these results established that gB, gL and gH/L epitopes were more accessible on Bo10 mutant virions than on WT or revertant viruses, consistent with gp180 hiding key epitopes from neutralization.

### Increased susceptibility of Bo10- BoHV-4 to gL directed neutralization

**Figure 8:**
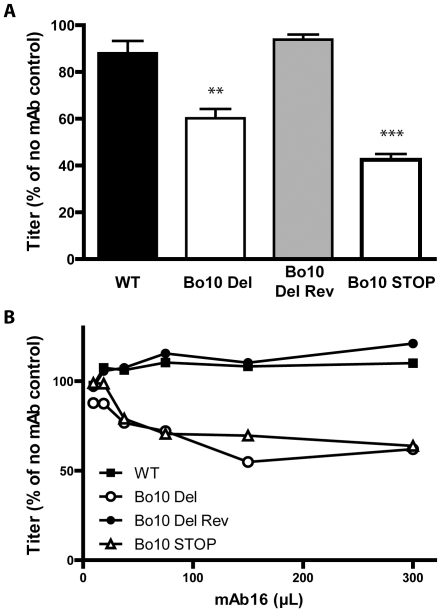
Sensitivity of Bo10- mutants to anti gL directed neutralization. **A**. BoHV-4 WT V.test, Bo10 del, Bo10 Rev and Bo10 STOP virions were incubated with the gL-specific neutralizing mAb 16. After incubation (2 h, 37°C) the viruses were plaque assayed for infectivity on MDBK cells. BoHV-4 titers are expressed relative to virus without antibody. The data presented are the average ± SEMs for triplicate measurements and were analyzed by 1way ANOVA and Bonferroni posttests, ** p<0.01, *** p<0.001. **B**. BoHV-4 WT V.test, Bo10 del, Bo10 Rev and Bo10 STOP virions were incubated with increasing amounts of the gL-specific neutralizing mAb 16, and then assayed for infectivity on MDBK cells. BoHV-4 titers are expressed relative to virus without antibody. Equivalent data were obtained in two further experiments.

We next tested whether mAbs recognizing Bo10 mutants better (Figure 6 and 7) could also neutralize them better than WT or revertant virions. While mAbs 29, 35 (anti-gB) and 33 (anti-gH/L) did not neutralize any strain (data not shown), mAb 16 (anti-gL) neutralized the Bo10 mutants better in different cell types (Figure 8A, Figure S5). It was not possible to achieve complete neutralization as it had been with immune sera (Figure 8B). Therefore, gL is likely to be only one of several neutralization targets protected by gp180 or other protection mechanisms exist. However, it was clearly one such target, establishing that the reduction in gL accessibility by gp180 was functionally important.

### Immunogenicity of BoHV-4 lacking gp180

**Figure 9:**
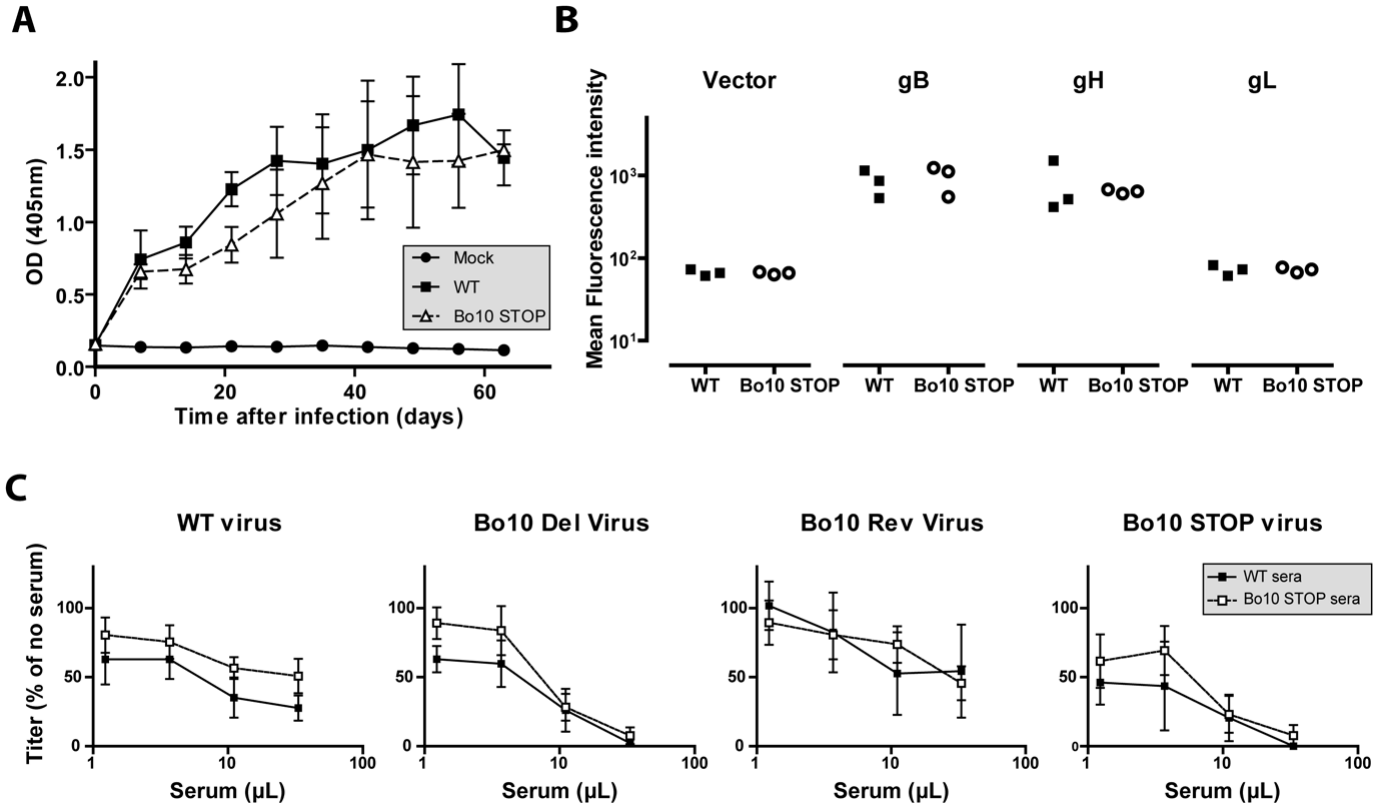
Immunogenicity of BoHV-4 lacking gp180. Rabbit anti-BoHV-4 V.test WT antibody response. Groups consisting of 3 rabbits were infected with 10^7^ PFU of BoHV-4 WT V.test and Bo10 STOP excised strains. **A**. Sera were collected at different times post-infection and the titre of anti-BoHV-4 antibodies was estimated by ELISA as described in the Material and Methods. Each value represents the mean +/- SD of the data obtained for the three rabbits of each group. The serum of a mock infected rabbit was taken as control. The data were analyzed by 2way ANOVA and Bonferroni posttests. No significative difference was observed between groups. **B**. Specific anti gB, gH and gL antibody responses were investigated by staining unfixed 293T cells expressing GPI-linked forms of gL, the gB extracellular domain or the gH extracellular domain. These staining were performed with sera collected 63 days post-infection. The data were analyzed by Student t-test. No significative difference was observed between WT and Bo10 STOP sera. **C**. BoHV-4 WT V.test, Bo10 Del, Bo10 Rev and Bo10 STOP virions were incubated with sera of WT or Bo10 STOP infected rabbits collected 63 days post-infection (3 sera per group). After incubation (2 h, 37°C) the viruses were plaque assayed for infectivity on MDBK cells. BoHV-4 titers are expressed relative to virus without antibody. The data presented are the average ± SEMs for 3 measurements and were analyzed by 2way ANOVA and Bonferroni posttests. No significative difference was observed between WT and Bo10 STOP sera.

The results obtained above showed that removal of gp180 results in the unmasking of several viral envelope epitopes among which some neutralization targets. To test whether gp180 might also affect BoHV-4 immunogenicity, we compared the humoral immune response induced in the rabbits by the Bo10 STOP strain to that observed with the wild type parental strain (Figure 9). Over the course of infection, no difference in total anti-BoHV-4 antibody response was observable between the groups of infected rabbits (Figure 9A). However, as the anti-herpesvirus antibody response is often dominated by capsid proteins, some subtle changes could be masked. We therefore investigated specific responses against gB, gH and gL. 293T cells expressing GPI-linked forms of gL, the gB extracellular domain or the gH extracellular domain, were stained with anti-BoHV-4 WT sera or with anti-BoHV-4 Bo10 STOP sera. The results obtained showed that sera of both groups of rabbits stained similarly gB and gH, whereas no detectable gL staining was observed (Figure 9B) although specific monoclonal antibodies confirmed cell surface expression of all proteins (data not shown). Finally, we compared the neutralization potential of these sera against WT, Bo10 Del, Bo10 STOP or Bo10 Rev virions. As observed previously for anti-BoHV-4 WT serum, anti-BoHV-4 Bo10 STOP serum neutralized Bo10 mutant viruses better. However, no significative difference in neutralization potential was observable between both groups of serum (Figure 9C). Our results suggest therefore that gp180 deficient virions display enhanced susceptibility to neutralizing antibodies but do not elicit markedly enhanced antibody response in infected rabbits. Thus, antigenicity does not predict immunogenicity.

### Gp180 O-glycans can be a target for neutralization

**Figure 10:**
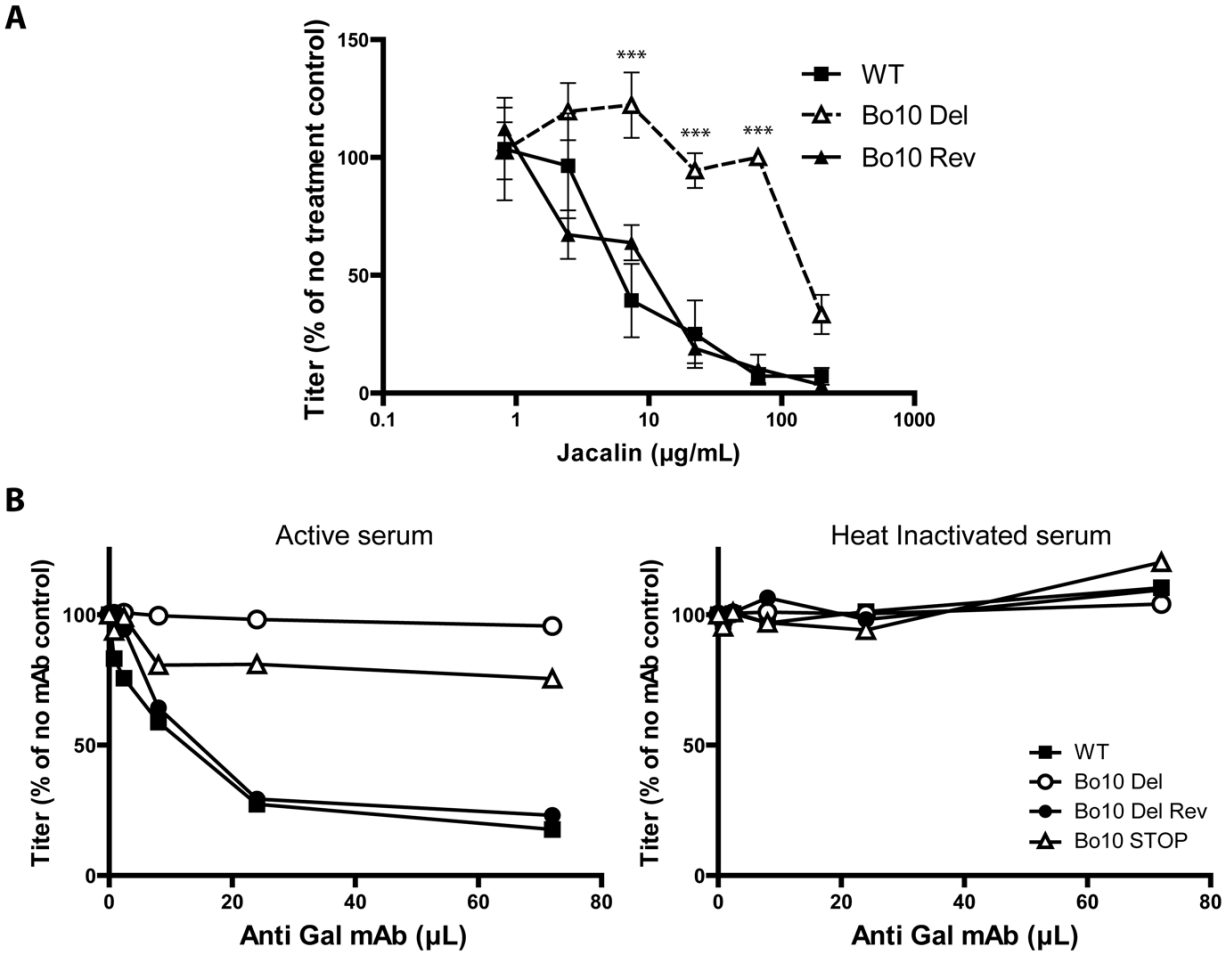
Glycans on BoHV-4 gp180 allow virus neutralization. **A**. Sensitivity of WT, Bo10 Del and Bo10 Rev strains to Jacalin mediated neutralization. BoHV-4 WT V.test, Bo10 Del and Bo10 Rev virions were incubated with increasing amounts of O-glycan specific lectin jacalin (1 h, 37°C) and then assayed for infectivity on MDBK cells. BoHV-4 titers are expressed relative to virus without lectin. The data presented are the average ± SEMs for 3 measurements and were analyzed by 2way ANOVA and Bonferroni posttests, *** p<0.001. **B**. Sensitivity to anti-Gal antibodies induced complement dependent neutralization of BoHV-4 WT, Bo10 Del, Bo10 Rev and Bo10 STOP strains. Virions of the different strains derived from MDBK cells were assayed for their sensitivity to neutralization by horse serum supplemented with increasing amounts of mAb M86 raised against Galα1-3Gal. Horse sera (heat inactivated or not by incubation at 56°C for 30 min) were tested at the final concentration of 10% (vol/vol). After incubation (2 h, 37°C) the viruses were plaque assayed for infectivity on MDBK cells. BoHV-4 titers are expressed relative to virus without antibody.

While O-glycans help BoHV-4 to evade neutralizing antibodies, they can potentially be targeted by carbohydrate binding agents, as proposed for other viruses. Gp180 is not essential for BoHV-4 replication, but lectins could still compromise virus entry by steric hindrance. We therefore tested the capacity of jacalin, an O-glycan-specific lectin, to inhibit BoHV-4 infection (Figure 10A). Inhibition was evident for WT and Bo10 Rev virions, whereas Bo10 deleted virions were relatively resistant. Therefore O-glycan-directed neutralization was possible for BoHV-4 and appeared to target mainly gp180.

Another strategy would be to use specific antibodies, much as HIV can be neutralized by an antibody that binds to the high-mannose glycans of its gp120 “silent face” [44]. In animals apart from humans, apes and Old World monkeys, the α1-3-galactosyltransferase enzyme adds a terminal galactose onto glycoproteins and glycolipids in a specific α1-3 linkage to generate the Gal epitope [45]. We have previously shown that human sera consequently exhibit innate BoHV-4 neutralization through complement activation by anti-Gal antibodies [46]. We therefore compared the sensitivity BoHV-4 WT, Bo10 Del, Bo10 Rev and Bo10 STOP virions to anti-Gal dependent neutralization. While complement-containing horse serum supplemented with anti-Gal antibodies neutralized WT and Bo10 Rev virions in a dose-dependent manner, Bo10 Del and Bo10 STOP virions were only slightly affected (Figure 10B). Thus gammaherpesvirus glycan shields are potentially accessible to neutralization by carbohydrate-specific antibodies.

## Discussion

Persistent viruses must evade multiple arms of the host immune response to maintain infectivity [47]–[48]. Gammaherpesviruses are archetypal persistent viruses, and their cytotoxic T cell evasion mechanisms are well-known [49]–[51]. Much less is known about how they evade neutralizing antibodies. Gammaherpesviruses all share a major glycoprotein homologous to EBV gp350. EBV remains infectious despite the presence of anti-gp350 antibodies in serum and saliva [52]–[55]. Moreover immunization with gp350 fails to reduce either infection rates or virus shedding [27]–[28]. Therefore, we still have much to learn about the interplay between gp350, gp350-specific antibodies and EBV host entry. For example, the inhibition of B cell infection by gp350-specific antibodies [54], [56] could have limited relevance to host entry, or even promote it by enhancing epithelial infection [26]. Similarly, antibodies to the MuHV-4 gp150 strongly enhance infection via IgG Fc receptors [57]. Here we showed that BoHV-4 gp180 is dispensable for establishment and maintenance of latency *in vivo* (Figure 2), but drastically reduced the susceptibility of BoHV-4 virions to neutralization by immune serum on various cell types (Figure 3, Figure S1). Gp180 seemed to hide at least partially several different epitopes on gB, gH and gL (Figures 6, 7 and S4), which included neutralization targets (Figures 8 and S5). Gp180 is extensively O-glycosylated and O-glycans account for most of its mass (Figures 4 and 5). These results suggest therefore that gp180 O-glycans provide part of a glycan shield for otherwise vulnerable viral epitopes. Since extensive O-glycosylation is a common feature of gammaherpesvirus gp350 homologs, this evasion mechanism may be widely shared. Another common feature of some of these proteins is strong immunogenicity [20], [23], [58]. The reason is not fully understood, however if gp180 homologs shield other virion glycoproteins, their location at the viral surface could favor development of an antibody response against them.

The substantial gp180 divergence between different BoHV-4 strains (Table S1, Figure 4A) remains to be explained. It is possible that much of the protein does not require a very specific amino acid sequence for its function. Thus, a key feature of the gp350 homologs of different gammaherpesviruses may simply be that they are type I transmembrane proteins with extensive O-glycosylation [33] (Figures 4 and 5A). An importance of glycans for immune evasion has also been hypothesized for gp350 [59]. Similarly, the HIV gp120 [60] uses glycans to provide a “silent face” protected against most antibodies [41]. Thus while neutralization is possible [61]–[62], this and other mechanisms ensure that it is difficult. The Ebola virus glycoprotein (EBOV GP) - again involved in virus binding [63] and a target for vaccine design - is also extensively glycosylated and in this way partially protected against antibody [64]. As with gp180, different filoviruses show huge glycoprotein diversity but retain the basic protein organization and extensive glycosylation [64]. This immune evasion mechanism appears therefore to be shared by several viral families.

While carbohydrates on SIV and HIV envelope proteins can shield these viruses from antibody recognition and neutralization [41], [65], it appears that these glycans could also limit the neutralizing antibody response in the context of SIV infection [66] or HIV immunization [67]. We did not observe an increased BoHV-4 immunogenicity in the absence of gp180 (Figure 9). While most of the epitopes hidden by the gp120 glycan shield are located on gp120 itself [68], BoHV-4 gp180 protects other virion glycoproteins in *trans*, rather than simply protecting itself in *cis*. Studies on different viruses [69]–[70] suggest that protection of a limited number of entry complexes from neutralization is probably sufficient to preserve virion infectivity. In contrast, influence on immunogenicity probably requires covering of all entry complexes in order to render them invisible to the immune system. Gp180 does not hide all the vulnerable epitopes at the viral surface. Indeed, even if gp180 hide most of the epitopes recognized by mAb 16, 29, 33 and 35, some remains accessible at the surface of WT or revertant virions (Figure 7). Moreover, infected cell debris provides also certainly a source of uncovered antigens. It therefore appears that gp180 influence virion antigenicity but not immunogenicity. Similarly, BoHV-4 gB N-term protects some vulnerable epitopes, but its deletion does not result in an enhanced ability to induce neutralizing antibody responses [71].

Another unusual feature of gp180 was the likely importance of O-linked glycans for viral antibody evasion. In other viruses, most protection against antibody seems to involve N-linked glycans [41], [72]. While BoHV-4 surface N-glycans are also involved in antibody evasion (Figure 5B), this study strengthens the role of O-glycans in neutralization evasion. O-linked glycans have also been shown to protect MuHV-4 gB N-terminal ectodomain against antibody [71]. Indeed, although MuHV-4 gB N-term confers protection to some neutralization epitopes on gH/L, gB N-term is itself a neutralization target [19], [71]. However, depending on the host cell, this part of gB can be largely protected against antibody by O-linked glycans [71]. These glycans could also possibly assist in protecting a neutralization epitope on gH/L. In BoHV-4, gB is the only other described envelope protein that could bear O-glycans. Indeed, gp180 and gB N-term contain respectively 122 and 33 predicted O-glycosylation sites [37]. These two proteins and their glycans could therefore cooperate to render BoHV-4 particularly resistant to neutralization [73]. Similar N-terminal O-glycans occur in the Herpes Simplex virus gC [74], but do not have a known function. Because N-linked glycans are relatively bulky, O-linked glycans may be better suited to protecting small or linear glycoprotein domains while still allowing protein/protein interactions. It seems with gp180 that protection by O-linked glycans can also be “scaled up” for more extensive protection. Because gp180 is likely to be part of a multi-protein complex, too many N-glycans might disrupt important protein/protein interactions. Another consideration is that glycans can on occasion be targeted by the immune response [75]. In this context, glycan diversity might be useful for a virus, and providing such diversity is a potential function of the BoHV-4 Bo17 gene, which encodes a mucin-type beta-1,6-N-acetylglucosaminyltransferase [76].

While glycans offer mainly protection in the natural setting, they can also be artificially targeted for neutralization by carbohydrate binding agents (CBAs) [77]. Evading CBAs would require a virus to compromise its glycan shield, thereby promoting neutralization by antibody [78]. As CBAs might be expected to elicit their own antibody response after repeated dosing, thereby attenuating their effect, anti-carbohydrate antibodies might be more useful in long-term settings. This also opens the possibility of vaccination against specific pathogen carbohydrates to target their glycan shields [79].

All together, our results suggest that BoHV-4 gp180 and, by extension, its homologs in other gammaherpesviruses shield the virus from immune recognition. This probably contributes to the ineffectiveness of the antibody response against these viruses.

## Materials and Methods

### Ethics statement

The experiments, maintenance and care of rabbits complied with the guidelines of the European Convention for the Protection of Vertebrate Animals used for Experimental and other Scientific Purposes (CETS n° 123). The protocol was approved by the Committee on the Ethics of Animal Experiments of the University of Liège, Belgium (Permit Number: 1035). All efforts were made to minimize suffering.

### Cells and virus

Madin-Darby bovine kidney (MDBK) (ATCC CCL-22), Bovine Turbinates (BT) (ATCC CRL-1390), Embryonic Bovine Trachea (EBTr) (ATCC CCL-44), Embryonic Bovine Lung (EBL) (DSMZ ACC-192), Bovine Macrophages (BOMAC) [80], bovine mammary epithelial (MacT) [81] and EBL-NLS-Cre [30] cells were cultured in Dulbecco’s modified Eagle Medium (Invitrogen) containing 10% fetal calf serum (FCS), 2% Penicillin/Streptomycin (Invitrogen) and 1% non Essential amino acids (Invitrogen). Bovine PBMC were prepared as described elsewhere [82] and cultured in RPMI Glutamax Medium containing 10% FCS, 2% Penicillin/Streptomycin (Invitrogen), 1% Essential amino acids (Invitrogen), 1 mM Sodium pyruvate, 25 mM HEPES and 50 µM 2-mercaptoethanol. The BoHV-4 V.test strain initially isolated from a case of orchitis [83], the BoHV-4 WTeGFP, Bo10 Del and Bo10 Rev strains [33] and the derived recombinant strain cloned as an Bacterial Artificial Chromosome (BAC) [30], were used throughout.

### Plasmids

The coding sequence for BoHV-4 V.test gL amino acid residues 1-140 was amplified by PCR (Hi-Fidelity PCR kit, Roche Diagnostics Ltd) with 5' *Avr*II-restricted and 3' *Not*I-restricted primers. Similarly, the coding sequences for BoHV-4 V.test gB amino acid residues 1–725 and gH amino acid residues 1–678 were amplified by PCR with 5' *Xba*I-restricted and 3' *Not*I-restricted primers. These PCR products were cloned into the *Xba*I/*Not*I sites of pBRAD, thereby attaching a C-terminal glycosyl-phosphatidyl-inositol (GPI) membrane anchor [20], generating gL-GPI, gB-GPI and gH-GPI expression plasmids.

### Antibodies and reagents

The O-glycan specific lectin, jacalin, was purchased from Vector Laboratories. For detection of gp180 on western blotting, we used a rabbit monospecific polyserum raised against the C-term end of the Bo10 encoded protein (anti-Bo10-c15) [33]. For the neutralization experiments, we used sera of 4 different rabbits infected intravenously with 10^8^ PFU of the BoHV-4 V.test strain and collected 63 days post inoculation. The mouse mAb M86 raised against the Galα1-3Gal epitope was purchased from Alexis and used free of sodium azide as previously described [46]. Horse serum was collected as a source of complement. The serum was treated as described previously to preserve complement activity, aliquoted and stored at −80°C [46].

Four mouse mAbs raised against BoHV-4 were also used in the present study [84]. Their specificities were unraveled on 293T cells transfected with the vectors encoding gB-GPI, gH-GPI or gL-GPI. The epitopes depending on the gH-gL heterodimer were reconstituted by co-expressing gH-GPI and gL-GPI. Briefly, transfected 293T cells were fixed and permeabilized in Acetone 95% for 5 min and then stained with the different antibodies in PBS containing 10% FCS (v/v). These antibodies were detected with Alexa 488-coupled Goat anti-mouse IgG-specific antibodies (Invitrogen). Nuclei were counterstained with DAPI (4,6-diamidino-2-phenylindole). Fluorescence was visualized with a Nikon TE-2000 microscope and a Leica CCD camera.

### Production of the BoHV-4 Bo10 STOP and Bo10 STOP Rev strains

We disrupted the BoHV-4 V.test Bo10 coding sequence (genomic coordinates 65,696 to 66,595, Genbank JN133502) by introducing stop codons into the coding sequence for the Bo10 signal peptide (Bo10 STOP). BoHV-4 recombinants were produced using BAC cloning and prokaryotic recombination technologies as described before [30]. The V.test BAC G plasmid was used as parental plasmid [30]. The BoHV-4 V.test Bo10 STOP was produced using a two step galactokinase (*gal*K) positive/negative selection in bacteria [85]. The first recombination process (*gal*K positive selection) consisted to introduce the galK gene into the Bo10 coding sequence (genomic coordinate 65,760) resulting in the V.test BAC G Bo10 *gal*K plasmid. Recombination was achieved using the Bo10 *gal*K cassette. It consisted of the *gal*K gene flanked by 50-bp sequences corresponding to Bo10 regions (65,711-65,760 and 65,810-65,761 of the BoHV-4 V.test strain genome). This cassette was produced by PCR using p*gal*K vector [85] as template and Bo10-fwd-*gal*K 5’agatctgtcatacattcaaattgc**atg**ctttttatattcagcctcgcctgCCTGTTGACAATTAATCATCGGCA 3’ and Bo10-rev-*gal*K 5’ atacggtggtggatgtgctggtgctgttgctggcagttaacccatatataTCAGCACTGTCCTGCTCCTT 3’ as forward and reverse primers, respectively (galK sequences are indicated in capital letters, Bo10 start codon is in bold). The second recombination process (*gal*K negative selection) consisted to replace the *gal*K sequence by a Bo10 STOP cassette to generate the BoHV-4 V.test Bo10 STOP plasmid. The Bo10 STOP cassette consisted of a synthetic double strand DNA corresponding to genomic coordinates 65,696 to 65,831 with the introduction (genomic coordinate 65,761) of 36 nucleotides coding for in-frame STOP codons and restriction sites (Figure 1A). These 36 nucleotides do not insert STOP codons in any of the 5 other frames of the genome. The BoHV-4 V.test Bo10 STOP Rev plasmid was produced similarly from BoHV-4 V.test Bo10 STOP plasmid. The first recombination process (*gal*K positive selection) was identical to the one described above. The second recombination process (*gal*K negative selection) consisted to restore Bo10 to generate a revertant plasmid. This cassette was produced by PCR using BoHV-4 V.test genome as template and Bo10-rec-sens (genomic coordinates 65,183 to 65,207) and Bo10-rec-rev (genomic coordinates 67,278 to 67,257) as forward and reverse primers, respectively. Reconstitution of infectious virus from BAC plasmids was obtained by transfection in MDBK cells to obtain Bo10 STOP BAC and Bo10 STOP BAC Rev strains. To excise the BAC cassette, reconstituted viruses were propagated in EBL-NLS-Cre cells expressing Cre recombinase to generate the corresponding excised strain.

### Southern blot

Southern blot analysis [82] of viral DNA digested with *Eco*RI was performed with probe corresponding to genomic coordinates 65,696 to 66,595 of the BoHV-4 V.test genome.

### Virus purification

BoHV-4 strains grown on MDBK cells were purified as follows. Virions were harvested from infected MDBK cell supernatants by ultracentrifugation (100,000× *g*, 2 h); infected-cell debris was then removed by low-speed centrifugation (1,000× *g*, 10 min). Virions were then centrifuged through a 20 to 50% (w/v) potassium tartrate gradient in PBS (100,000× *g*, 2 h). Virions were recovered from the gradient and finally washed and concentrated in PBS (100,000× *g*, 2 h).

### Western blot

Virions were lysed and denatured by heating (95°C, 5 min) in SDS-PAGE sample buffer (31.25 mM Tris-HCl pH 6.8, 1% (w/v) SDS, 12.5% (w/v) glycerol, 0.005% (w/v) Bromophenol Blue, 2.5% (v/v) 2-mercaptoethanol). Proteins were resolved by electrophoresis on Mini-PROTEAN TGX (Tris-Glycine eXtended) precast 7.5% resolving gels (Bio-Rad) in SDS-PAGE running buffer (25 mM Tris-base, 192 mM glycine, 0.1% (w/v) SDS) and transferred to polyvinylidene difluoride membranes (Immobilon-P transfer membrane, 0.45 µM pore size, Millipore). The membranes were blocked with 3% non-fat milk in PBS/0.1% Tween-20, and then incubated with anti-Bo10-c15 rabbit antibodies, mAb 35 or rabbit anti-BoHV-4 polyserum in the same buffer. Bound antibodies were detected with horseradish peroxidase-conjugated goat anti-rabbit IgG pAb or goat anti-mouse IgG pAb (Dako Corporation), followed by washing in PBS/0.1% Tween-20, development with ECL substrate (GEHealthcare) and exposure to X-ray film.

### Animals

Specific-pathogen-free New-Zealand white rabbits were used throughout this study. Rabbits were inoculated intravenously with purified stocks of the different viral strains. In one experiment we infected rabbits with WT, Bo10 Del or Bo10 Rev strains (10^8^ PFU). In a second experiment, rabbits received WT or Bo10 STOP strains (10^7^ PFU). At the end of the experiment, rabbits were euthanized and a necropsy examination was performed during which the spleen was collected.

### Isolation of peripheral blood mononuclear cells and preparation of spleen cell suspension

Blood samples were collected and PBMC were separated by Ficoll (Ficoll-Paque Plus, GE Healthcare) density gradient as described previously [86]. Immediately after euthanasia, spleen was removed and half-part of it was homogenized using a tissue grinder (VWR), passed through a stainless steel sieve and washed in FCS-free MEM before further analyses.

### Viral genome detection by Real time-PCR

DNA was purified from the spleen and PBMC using the QIAamp DNA Mini kit (Qiagen). Real-time PCR was performed as described elsewhere [87]. A 103 bp fragment corresponding to BoHV-4 ORF8 was amplified with the forward primer 8startfw (5’- CAAATAGTTCATTAGCTGCCTCTCC -3’) and the reverse primer 8middlerev (5’- TCATCAGTAACAGTTGGAATAGTGG -3’) in the presence of the fluorescent probe 5’-FAM-AACACGTCAACA AGCAAGCCATCCACTG-TAMRA-3’. pGEM-T easy containing BoHV-4 gB ORF was used to establish standard curves. PCR amplifications and fluorescence reactions were carried out in a iCycler system (Bio-Rad) under the following conditions: initial activation of the *Taq* polymerase (Bio-Rad) at 94°C for 5 min followed by 50 cycles at 94°C for 1 min, 50 cycles at 51°C for 30 sec and 50 cycles at 72°C for 1 min.

### Virus detection by infectious centre assay

Viral detection in spleen cell suspension was assayed by infectious centre assay (ICA) as follows. 5.10^5^ MDBK cells grown in 6 well cluster dishes (Becton Dickinson) were co-cultured for 7 days at 37°C with spleen cells in MEM containing 10% FCS, 2% PS, 0.6% CMC and 5.10^−5^M of β-mercaptoethanol (Merck). Cells were then fixed and stained with mAb 35 for indirect immunofluorescent detection of intracellular viral antigen as described previously [33]. Fluorescence was then visualized with a TE2000-S Nikon and a Leica DC300F CCD camera system.

### Oligosaccharide digestion

All reagents were obtained from New England BioLabs. For SDS-PAGE analysis, samples were denatured in Glycoprotein Denaturing Buffer (0.5% SDS, 40 mM DTT) for 10 min at 100°C and then digested for 3 h at 37°C with 250 NEB units PNGase F and/or 250 NEB units of neuraminidase, β1-4 Galactosidase, O-glycanase in G7 reaction buffer (50 mM sodium phosphate, pH 7.5) with 1% NP-40. Reactions were stopped by the addition of Laemmli sample buffer and proteins were analyzed by immunoblotting as described below. For neutralization assays, N- or O-linked glycans were removed with the same enzymes, but without reduction or denaturation. Thus, intact virions were incubated with the different enzymes (3 h, 37°C) in PBS/5% fetal calf serum buffered to pH 6.

### Flow cytometry

For cell surface staining, cells infected by the different virus strains (2 PFU/cell, 36 h) were washed in PBS and analyzed directly for green channel fluorescence [71]. For intracellular staining, cells were fixed in 1% paraformaldehyde (30 min at room temperature) and then permeabilized with 0.1% saponin. Cells were incubated (1 h, 4°C) with the different mAbs specific for BoHV-4 glycoproteins followed by Alexa 633-conjugated goat anti-mouse pAb (Invitrogen). Cells were then washed and analyzed on a FACSAria cytometer (Becton Dickinson).

### Immunogold labeling of virions

Copper grids of 400 mesh (Agar Scientific Ltd) were incubated for 10 min with 2% Alcian blue 8G solution (Gurr Microscopy Materials, BHD) to add positive charges. After washing, purified virions (10^8^ PFU/ml) were adsorbed to the grids for 10 min. Non-specific interactions were blocked by incubation of the grids for 15 min in PBS containing 0.1% (w/v) cold water fish skin gelatin (CWFG, Aurion) and 5% (w/v) goat serum (Invitrogen). This solution was also used for further incubation and washes. Immunogold labeling was performed by incubation of the grids with mAb 35 as primary antibody for 60 min at room temperature. After washing with PBS and incubation in PBS 0.1% CWFG 5% goat serum for 5 min, the grids were incubated with Goat anti-mouse IgG-10 nm gold labeled conjugate (diluted 1∶50, BBInternational) as secondary antibody for 60 min at RT. A final incubation step was performed in 2% uranyl acetate solution for 10 sec (Agar Scientific). Immunogold stained virions were observed using a transmission electron microscope (FEI, TEcnai Biotwin). Micrographs of virion were acquired for at least 20 individual virions per strain.

### Indirect immunofluorescent staining of bound virions

Infected cells (20 PFU/cell, 2 h, 4°C) were fixed in cold Acetone 95% for 5 min on ice. Immunofluorescent staining (incubation and washes) was performed in PBS containing 10% FCS (v/v). Samples were incubated at RT for 45 min with the different mAbs raised against BoHV-4 glycoproteins. After three washes, samples were incubated at RT for 45 min with Alexa Fluor 488 or Alexa Fluor 568 goat anti-mouse IgG (2 µg/ml; Invitrogen). Images were acquired on a Leica TCS SP confocal laser scanning microscope with settings specific for Alexa Fluor 488 or Alexa Fluor 568. Acquisition settings (PMT voltage and offset) were kept identical between slides stained with the same antibodies.

### Quantification of anti-BoHV-4 antibodies by ELISA

Nunc Maxisorp ELISA plates (Nalgene Nunc) were coated for 18 h at 37°C with 0.1% Tween 20-disrupted BoHV-4 virions (2.10^6^ PFU/well), blocked in PBS/0.1% Tween-20/3% BSA, and incubated with rabbit sera (diluted 1/300 in PBS/0.1% Tween-20/3% BSA). Bound antibodies were detected with Alkaline Phosphatase conjugated goat anti-rabbit Ig polyclonal antibody (Sigma). Washing were performed with PBS/0.1% Tween-20/3% BSA. p-Nitrophenylphosphate (Sigma) was used as substrate and absorbance was read at 405 nm using a Benchmark ELISA plate reader (Thermo).

### Nucleotide sequence accession numbers

Sequence data reported here have been deposited in the GenBank database under the following accession numbers: **BoHV-4 V.test Long Unique region** (JN133502).

## Supporting Information

Figure S1 **Neutralization of the BoHV-4 gp180 deficient virions on various cell types.** BoHV-4 V.test WTeGFP and Bo10 Del virions were incubated with serum of a rabbit infected with BoHV-4 V.test strain. After incubation (2h, 37°C) the viruses were used to infect MDBK, BT, EBTr, EBL, BOMAC and MacT cells. BoHV-4 titers are expressed relative to virus without antibody.
(TIF)

Figure S2 **Effect of deglycosylation treatments on BoHV-4 infectivity.** Intact BoHV-4 V.test WT virions were deglycosylated without denaturation as described in the Material and Methods. N-, PNGase F; O-, neuraminidase + β1-4 Galactosidase + O-glycanase; NO-, PNGase F + neuraminidase + β1-4 Galactosidase + O-glycanase. After deglycosylation treatment, virion samples were titered and these titers were expressed as percentage of the titers measured before deglycosylation.
(TIF)

Figure S3 **Identification of the targets of mAbs 16, 29 and 33.** In order to map mAb recognition, 293T cells were transfected with the gB or gH extracellular domains or the entire gL fused to a GPI membrane anchor resulting in gB-GPI, gH-GPI and gL-GPI respectively. To reconstitute epitopes depending on the gH-gL hetrodimer, we cotransfected the cells with plasmids encoding gH-GPI and gL-GPI. Forty-eight hours after transfection, the cells were fixed and stained with the different mAbs as indicated.
(TIF)

Figure S4 **Immunoelectron microscopy of virions.** Purified BoHV-4 WT V.test or Bo10 Del virions were processed for immuno-electron microscopy as described in the Material and Methods. These samples were stained for gB with mAb 35 followed by secondary goat anti-mouse IgG-10 nm gold labeled. Pictures of individual virions were then taken. Pictures of 5 representative virions per strain are showed.
(TIF)

Figure S5 **Sensitivity of the Bo10 STOP strain to anti gL directed neutralization. A. and B.** BoHV-4 V.test WT BAC, Bo10 STOP and Bo10 STOP Rev virions were incubated with the gL-specific neutralizing mAb 16 (100 µg/mL). After incubation (2h, 37°C) the viruses were plaque assayed for infectivity on either MDBK cells (A) or EBL cells (B). After 4 days of incubation, the plates were fixed and the plaques were counted. BoHV-4 titers are expressed relative to virus without antibody. The data presented are the average ± SEMs for triplicate measurements and were analyzed by 1way ANOVA and Bonferroni posttests, * p<0.05, ** p<0.01, *** p<0.001.
(TIF)

Table S1 **Bo10 sequences divergences among BoHV-4 strains.** Nucleotide and amino acid sequences divergences of Bo10 genes from 9 BoHV-4 strains were determined using CLUSTALw. Values above and below the diagonal refer to percentages of nucleotide and amino acid sequences divergences, respectively.
(DOC)

## References

[1] Thorley-Lawson DA, Gross A (2004). Persistence of the Epstein-Barr virus and the origins of associated lymphomas.. N Engl J Med.

[2] Verma SC, Robertson ES (2003). Molecular biology and pathogenesis of Kaposi sarcoma-associated herpesvirus.. FEMS Microbiol Lett.

[3] Salvetti M, Giovannoni G, Aloisi F (2009). Epstein-Barr virus and multiple sclerosis.. Curr Opin Neurol.

[4] Serafini B, Rosicarelli B, Franciotta D, Magliozzi R, Reynolds R (2007). Dysregulated Epstein-Barr virus infection in the multiple sclerosis brain.. J Exp Med.

[5] Hadinoto V, Shapiro M, Sun CC, Thorley-Lawson DA (2009). The dynamics of EBV shedding implicate a central role for epithelial cells in amplifying viral output.. PLoS Pathog.

[6] Niederman JC, Miller G, Pearson HA, Pagano JS, Dowaliby JM (1976). Infectious mononucleosis. Epstein-Barr-virus shedding in saliva and the oropharynx.. N Engl J Med.

[7] Yao QY, Rickinson AB, Epstein MA (1985). A re-examination of the Epstein-Barr virus carrier state in healthy seropositive individuals.. Int J Cancer.

[8] Desgranges C, de-The G (1978). Presence of Epstein-Barr virus specific IgA in saliva of nasopharyngeal carcinoma patients: their activity, origin and possible clinical value..

[9] Sarid O, Anson O, Yaari A, Margalith M (2001). Epstein-Barr virus specific salivary antibodies as related to stress caused by examinations.. J Med Virol.

[10] Buisman AM, Abbink F, Schepp RM, Sonsma JA, Herremans T (2008). Preexisting poliovirus-specific IgA in the circulation correlates with protection against virus excretion in the elderly.. J Infect Dis.

[11] Ogra PL, Karzon DT, Righthand F, MacGillivray M (1968). Immunoglobulin response in serum and secretions after immunization with live and inactivated poliovaccine and natural infection.. N Engl J Med.

[12] Burton DR (2002). Antibodies, viruses and vaccines.. Nat Rev Immunol.

[13] Heldwein EE, Krummenacher C (2008). Entry of herpesviruses into mammalian cells.. Cell Mol Life Sci.

[14] Chandran B (2010). Early events in Kaposi's sarcoma-associated herpesvirus infection of target cells.. J Virol.

[15] Hutt-Fletcher LM (2007). Epstein-Barr virus entry.. J Virol.

[16] Pertel PE (2002). Human herpesvirus 8 glycoprotein B (gB), gH, and gL can mediate cell fusion.. J Virol.

[17] Miller N, Hutt-Fletcher LM (1988). A monoclonal antibody to glycoprotein gp85 inhibits fusion but not attachment of Epstein-Barr virus.. J Virol.

[18] Gill MB, Gillet L, Colaco S, May JS, de Lima BD (2006). Murine gammaherpesvirus-68 glycoprotein H-glycoprotein L complex is a major target for neutralizing monoclonal antibodies.. J Gen Virol.

[19] Gillet L, Gill MB, Colaco S, Smith CM, Stevenson PG (2006). Murine gammaherpesvirus-68 glycoprotein B presents a difficult neutralization target to monoclonal antibodies derived from infected mice.. J Gen Virol.

[20] Gillet L, May JS, Colaco S, Stevenson PG (2007). The murine gammaherpesvirus-68 gp150 acts as an immunogenic decoy to limit virion neutralization.. PLoS One.

[21] Nemerow GR, Mold C, Schwend VK, Tollefson V, Cooper NR (1987). Identification of gp350 as the viral glycoprotein mediating attachment of Epstein-Barr virus (EBV) to the EBV/C3d receptor of B cells: sequence homology of gp350 and C3 complement fragment C3d.. J Virol.

[22] Tanner J, Weis J, Fearon D, Whang Y, Kieff E (1987). Epstein-Barr virus gp350/220 binding to the B lymphocyte C3d receptor mediates adsorption, capping, and endocytosis.. Cell.

[23] Thorley-Lawson DA, Poodry CA (1982). Identification and isolation of the main component (gp350-gp220) of Epstein-Barr virus responsible for generating neutralizing antibodies in vivo.. J Virol.

[24] Janz A, Oezel M, Kurzeder C, Mautner J, Pich D (2000). Infectious Epstein-Barr virus lacking major glycoprotein BLLF1 (gp350/220) demonstrates the existence of additional viral ligands.. J Virol.

[25] Shannon-Lowe CD, Neuhierl B, Baldwin G, Rickinson AB, Delecluse HJ (2006). Resting B cells as a transfer vehicle for Epstein-Barr virus infection of epithelial cells.. Proc Natl Acad Sci U S A.

[26] Turk SM, Jiang R, Chesnokova LS, Hutt-Fletcher LM (2006). Antibodies to gp350/220 enhance the ability of Epstein-Barr virus to infect epithelial cells.. J Virol.

[27] Sokal EM, Hoppenbrouwers K, Vandermeulen C, Moutschen M, Leonard P (2007). Recombinant gp350 vaccine for infectious mononucleosis: a phase 2, randomized, double-blind, placebo-controlled trial to evaluate the safety, immunogenicity, and efficacy of an Epstein-Barr virus vaccine in healthy young adults.. J Infect Dis.

[28] Cox C, Naylor BA, Mackett M, Arrand JR, Griffin BE (1998). Immunization of common marmosets with Epstein-Barr virus (EBV) envelope glycoprotein gp340: effect on viral shedding following EBV challenge.. J Med Virol.

[29] Nash AA, Dutia BM, Stewart JP, Davison AJ (2001). Natural history of murine gamma-herpesvirus infection.. Philos Trans R Soc Lond B Biol Sci.

[30] Gillet L, Daix V, Donofrio G, Wagner M, Koszinowski UH (2005). Development of bovine herpesvirus 4 as an expression vector using bacterial artificial chromosome cloning.. J Gen Virol.

[31] Zimmermann W, Broll H, Ehlers B, Buhk HJ, Rosenthal A (2001). Genome sequence of bovine herpesvirus 4, a bovine Rhadinovirus, and identification of an origin of DNA replication.. J Virol.

[32] de Lima BD, May JS, Stevenson PG (2004). Murine gammaherpesvirus 68 lacking gp150 shows defective virion release but establishes normal latency in vivo.. J Virol.

[33] Machiels B, Lete C, de Fays K, Mast J, Dewals B (2010). Bovine Herpesvirus-4 Bo10 gene encodes a non-essential viral envelope protein that regulates viral tropism through both positive and negative effects.. J Virol.

[34] Dewals B, Thirion M, Markine-Goriaynoff N, Gillet L, de Fays K (2006). Evolution of Bovine herpesvirus 4: recombination and transmission between African buffalo and cattle.. J Gen Virol.

[35] Shin YC, Jones LR, Manrique J, Lauer W, Carville A (2010). Glycoprotein gene sequence variation in rhesus monkey rhadinovirus.. Virology.

[36] Dunowska M, Letchworth GJ, Collins JK, DeMartini JC (2001). Ovine herpesvirus-2 glycoprotein B sequences from tissues of ruminant malignant catarrhal fever cases and healthy sheep are highly conserved.. J Gen Virol.

[37] Julenius K, Molgaard A, Gupta R, Brunak S (2005). Prediction, conservation analysis, and structural characterization of mammalian mucin-type O-glycosylation sites.. Glycobiology.

[38] Maley F, Trimble RB, Tarentino AL, Plummer TH (1989). Characterization of glycoproteins and their associated oligosaccharides through the use of endoglycosidases.. Anal Biochem.

[39] Skehel JJ, Stevens DJ, Daniels RS, Douglas AR, Knossow M (1984). A carbohydrate side chain on hemagglutinins of Hong Kong influenza viruses inhibits recognition by a monoclonal antibody.. Proc Natl Acad Sci U S A.

[40] Aguilar HC, Matreyek KA, Filone CM, Hashimi ST, Levroney EL (2006). N-glycans on Nipah virus fusion protein protect against neutralization but reduce membrane fusion and viral entry.. J Virol.

[41] Wei X, Decker JM, Wang S, Hui H, Kappes JC (2003). Antibody neutralization and escape by HIV-1.. Nature.

[42] Gillet L, Stevenson PG (2007). Evidence for a multiprotein gamma-2 herpesvirus entry complex.. J Virol.

[43] Lomonte P, Filee P, Lyaku JR, Bublot M, Pastoret PP (1997). Glycoprotein B of bovine herpesvirus 4 is a major component of the virion, unlike that of two other gammaherpesviruses, Epstein-Barr virus and murine gammaherpesvirus 68.. J Virol.

[44] Scanlan CN, Pantophlet R, Wormald MR, Ollmann Saphire E, Stanfield R (2002). The broadly neutralizing anti-human immunodeficiency virus type 1 antibody 2G12 recognizes a cluster of alpha1—>2 mannose residues on the outer face of gp120.. J Virol.

[45] Galili U, Clark MR, Shohet SB, Buehler J, Macher BA (1987). Evolutionary relationship between the natural anti-Gal antibody and the Gal alpha 1----3Gal epitope in primates.. Proc Natl Acad Sci U S A.

[46] Machiels B, Gillet L, Nascimento Brito SD, Drion P, Delforge C (2007). Natural antibody—complement dependent neutralization of bovine herpesvirus 4 by human serum.. Microbes Infect.

[47] Lavine JS, Poss M, Grenfell BT (2008). Directly transmitted viral diseases: modeling the dynamics of transmission.. Trends Microbiol.

[48] Villarreal LP, Defilippis VR, Gottlieb KA (2000). Acute and persistent viral life strategies and their relationship to emerging diseases.. Virology.

[49] Tortorella D, Gewurz BE, Furman MH, Schust DJ, Ploegh HL (2000). Viral subversion of the immune system.. Annu Rev Immunol.

[50] Yewdell JW, Hill AB (2002). Viral interference with antigen presentation.. Nat Immunol.

[51] Stevenson PG, Simas JP, Efstathiou S (2009). Immune control of mammalian gamma-herpesviruses: lessons from murid herpesvirus-4.. J Gen Virol.

[52] Hoffman GJ, Lazarowitz SG, Hayward SD (1980). Monoclonal antibody against a 250,000-dalton glycoprotein of Epstein-Barr virus identifies a membrane antigen and a neutralizing antigen.. Proc Natl Acad Sci U S A.

[53] Miller G, Heston L, Hoffman G (1982). Neutralization of lymphocyte immortalization by different strains of Epstein-Barr virus with a murine monoclonal antibody.. Infect Immun.

[54] Sashihara J, Burbelo PD, Savoldo B, Pierson TC, Cohen JI (2009). Human antibody titers to Epstein-Barr Virus (EBV) gp350 correlate with neutralization of infectivity better than antibody titers to EBV gp42 using a rapid flow cytometry-based EBV neutralization assay.. Virology.

[55] Moutschen M, Leonard P, Sokal EM, Smets F, Haumont M (2007). Phase I/II studies to evaluate safety and immunogenicity of a recombinant gp350 Epstein-Barr virus vaccine in healthy adults.. Vaccine.

[56] Miller G, Niederman JC, Stitt DA (1972). Infectious mononucleosis: appearance of neutralizing antibody to Epstein-Barr virus measured by inhibition of formation of lymphoblastoid cell lines.. J Infect Dis.

[57] Rosa GT, Gillet L, Smith CM, de Lima BD, Stevenson PG (2007). IgG fc receptors provide an alternative infection route for murine gamma-herpesvirus-68.. PLoS One.

[58] Chandran B, Smith MS, Koelle DM, Corey L, Horvat R (1998). Reactivities of human sera with human herpesvirus-8-infected BCBL-1 cells and identification of HHV-8-specific proteins and glycoproteins and the encoding cDNAs.. Virology.

[59] Szakonyi G, Klein MG, Hannan JP, Young KA, Ma RZ (2006). Structure of the Epstein-Barr virus major envelope glycoprotein.. Nat Struct Mol Biol.

[60] Johnson WE, Desrosiers RC (2002). Viral persistence: HIV's strategies of immune system evasion.. Annu Rev Med.

[61] Wu X, Yang ZY, Li Y, Hogerkorp CM, Schief WR (2010). Rational design of envelope identifies broadly neutralizing human monoclonal antibodies to HIV-1.. Science.

[62] Zhou T, Georgiev I, Wu X, Yang ZY, Dai K (2010). Structural basis for broad and potent neutralization of HIV-1 by antibody VRC01.. Science.

[63] Lee JE, Saphire EO (2009). Neutralizing ebolavirus: structural insights into the envelope glycoprotein and antibodies targeted against it.. Curr Opin Struct Biol.

[64] Lee JE, Fusco ML, Hessell AJ, Oswald WB, Burton DR (2008). Structure of the Ebola virus glycoprotein bound to an antibody from a human survivor.. Nature.

[65] Back NK, Smit L, De Jong JJ, Keulen W, Schutten M (1994). An N-glycan within the human immunodeficiency virus type 1 gp120 V3 loop affects virus neutralization.. Virology.

[66] Reitter JN, Means RE, Desrosiers RC (1998). A role for carbohydrates in immune evasion in AIDS.. Nat Med.

[67] Li Y, Cleveland B, Klots I, Travis B, Richardson BA (2008). Removal of a single N-linked glycan in human immunodeficiency virus type 1 gp120 results in an enhanced ability to induce neutralizing antibody responses.. J Virol.

[68] Pantophlet R, Burton DR (2006). GP120: target for neutralizing HIV-1 antibodies.. Annu Rev Immunol.

[69] Yang X, Kurteva S, Lee S, Sodroski J (2005). Stoichiometry of antibody neutralization of human immunodeficiency virus type 1.. J Virol.

[70] Pierson TC, Xu Q, Nelson S, Oliphant T, Nybakken GE (2007). The stoichiometry of antibody-mediated neutralization and enhancement of West Nile virus infection.. Cell Host Microbe.

[71] Gillet L, Stevenson PG (2007). Antibody evasion by the N terminus of murid herpesvirus-4 glycoprotein B.. EMBO J.

[72] Helle F, Vieyres G, Elkrief L, Popescu CI, Wychowski C (2010). Role of N-linked glycans in the functions of hepatitis C virus envelope proteins incorporated into infectious virions.. J Virol.

[73] Dubuisson J, Guillaume J, Boulanger D, Thiry E, Bublot M (1990). Neutralization of bovine herpesvirus type 4 by pairs of monoclonal antibodies raised against two glycoproteins and identification of antigenic determinants involved in neutralization.. J Gen Virol.

[74] Biller M, Mardberg K, Hassan H, Clausen H, Bolmstedt A (2000). Early steps in O-linked glycosylation and clustered O-linked glycans of herpes simplex virus type 1 glycoprotein C: effects on glycoprotein properties.. Glycobiology.

[75] Doores KJ, Fulton Z, Hong V, Patel MK, Scanlan CN (2010). A nonself sugar mimic of the HIV glycan shield shows enhanced antigenicity.. Proc Natl Acad Sci U S A.

[76] Vanderplasschen A, Markine-Goriaynoff N, Lomonte P, Suzuki M, Hiraoka N (2000). A multipotential beta -1,6-N-acetylglucosaminyl-transferase is encoded by bovine herpesvirus type 4.. Proc Natl Acad Sci U S A.

[77] Balzarini J (2007). Targeting the glycans of glycoproteins: a novel paradigm for antiviral therapy.. Nat Rev Microbiol.

[78] Balzarini J (2005). Targeting the glycans of gp120: a novel approach aimed at the Achilles heel of HIV.. Lancet Infect Dis.

[79] Astronomo RD, Burton DR (2010). Carbohydrate vaccines: developing sweet solutions to sticky situations?. Nat Rev Drug Discov.

[80] Donofrio G, van Santen VL (2001). A bovine macrophage cell line supports bovine herpesvirus-4 persistent infection.. J Gen Virol.

[81] Huynh HT, Robitaille G, Turner JD (1991). Establishment of bovine mammary epithelial cells (MAC-T): an in vitro model for bovine lactation.. Exp Cell Res.

[82] Gillet L, Schroeder H, Mast J, Thirion M, Renauld JC (2009). Anchoring tick salivary anti-complement proteins IRAC I and IRAC II to membrane increases their immunogenicity.. Vet Res.

[83] Thiry E, Pastoret PP, Dessy-Doizé C, Hanzen C, Calberg-Bacq CM (1981). Herpesvirus in infertile bull's testicle.. Vet rec.

[84] Dubuisson J, Thiry E, Bublot M, Sneyers M, Boulanger D (1989). Production and characterization of monoclonal antibodies to bovid herpesvirus-4.. Vet Microbiol.

[85] Warming S, Costantino N, Court DL, Jenkins NA, Copeland NG (2005). Simple and highly efficient BAC recombineering using galK selection.. Nucleic Acids Res.

[86] Dewals B, Boudry C, Farnir F, Drion PV, Vanderplasschen A (2008). Malignant catarrhal fever induced by alcelaphine herpesvirus 1 is associated with proliferation of CD8+ T cells supporting a latent infection.. PLoS ONE.

[87] Boudry C, Markine-Goriaynoff N, Delforge C, Springael JY, de Leval L (2007). The A5 gene of alcelaphine herpesvirus 1 encodes a constitutively active G-protein-coupled receptor that is non-essential for the induction of malignant catarrhal fever in rabbits.. J Gen Virol.

[88] Thompson JD, Gibson TJ, Plewniak F, Jeanmougin F, Higgins DG (1997). The CLUSTAL_X windows interface: flexible strategies for multiple sequence alignment aided by quality analysis tools.. Nucleic Acids Res.

[89] Bendtsen JD, Nielsen H, von Heijne G, Brunak S (2004). Improved prediction of signal peptides: SignalP 3.0.. J Mol Biol.

