# Supplementary figures and images for: Antibody Evasion by a Gammaherpesvirus O-Glycan Shield
Source: PLoS Pathog. 2011 Nov 17;7(11):e1002387. doi: 10.1371/journal.ppat.1002387 (PMC3219721; doi:10.1371/journal.ppat.1002387)

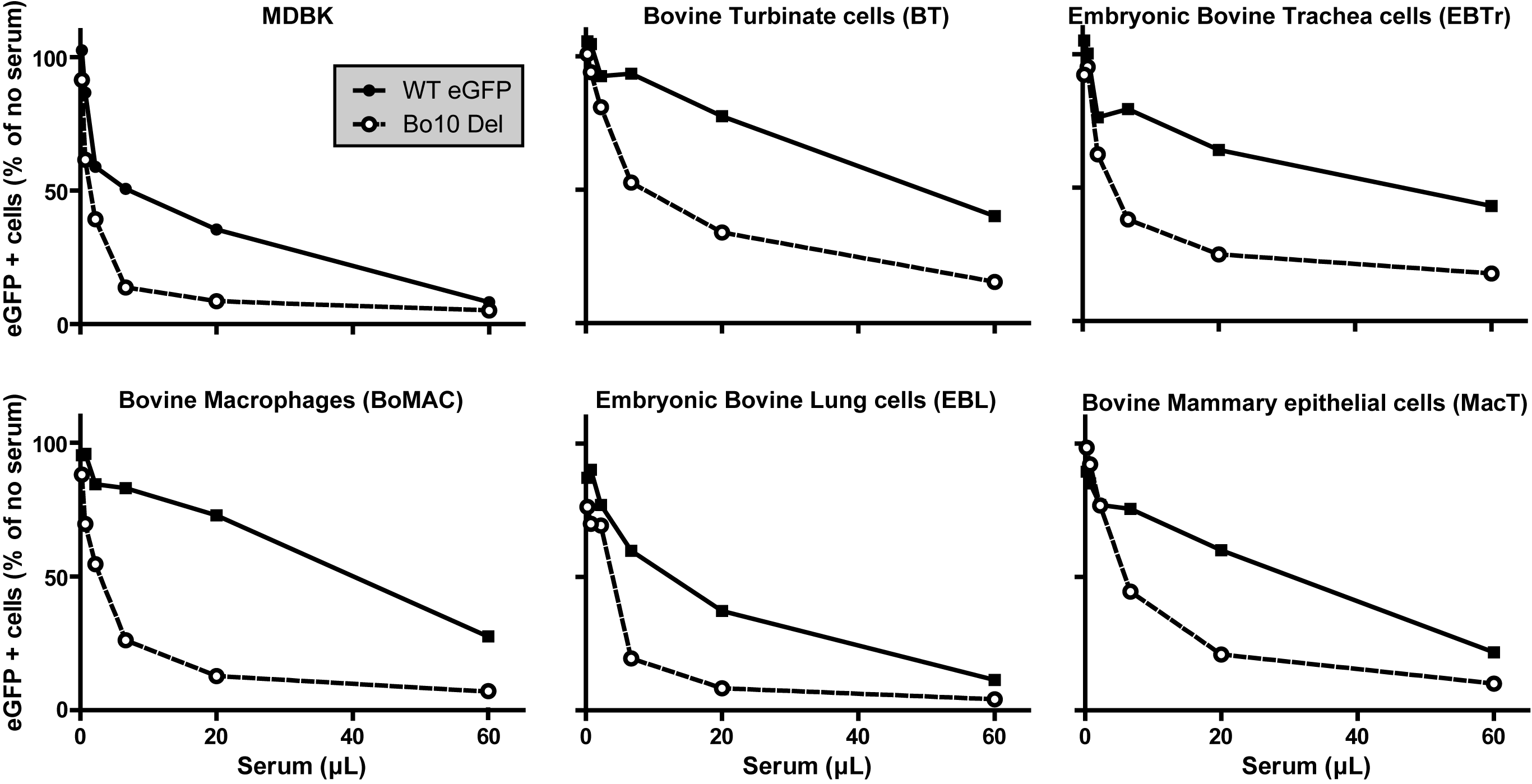

Supplement: Figure S1 — Neutralization of the BoHV-4 gp180 deficient virions on various cell types. BoHV-4 V.test WTeGFP and Bo10 Del virions were incubated with serum of a rabbit infected with BoHV-4 V.test strain. After incubation (2h, 37°C) the viruses were used to infect MDBK, BT, EBTr, EBL, BOMAC and MacT cells. BoHV-4 titers are expressed relative to virus without antibody. (TIF) [file ppat.1002387.s001.tif]

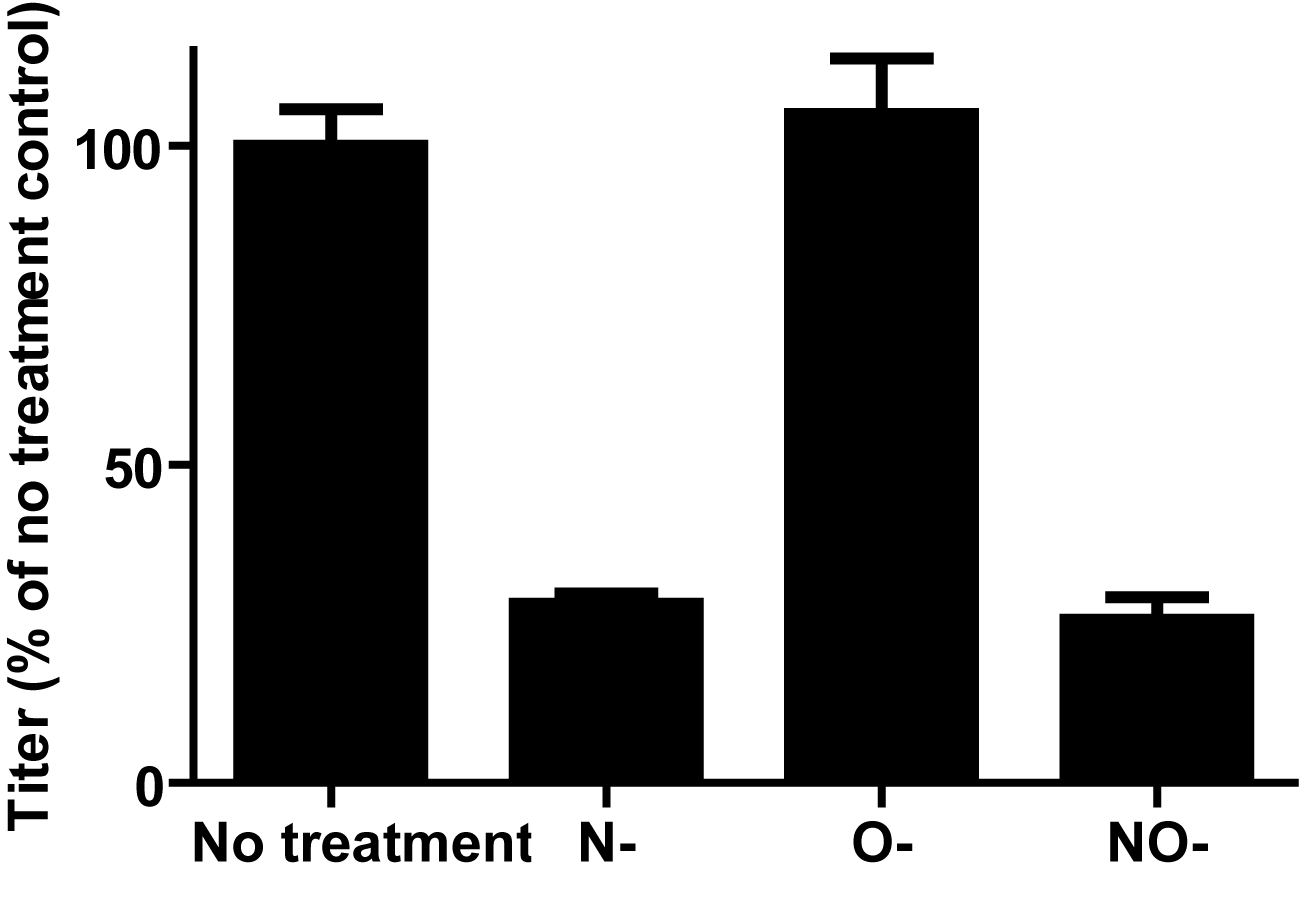

Supplement: Figure S2 — Effect of deglycosylation treatments on BoHV-4 infectivity. Intact BoHV-4 V.test WT virions were deglycosylated without denaturation as described in the Material and Methods. N-, PNGase F; O-, neuraminidase + β1-4 Galactosidase + O-glycanase; NO-, PNGase F + neuraminidase + β1-4 Galactosidase + O-glycanase. After deglycosylation treatment, virion samples were titered and these titers were expressed as percentage of the titers measured before deglycosylation. (TIF) [file ppat.1002387.s002.tif]

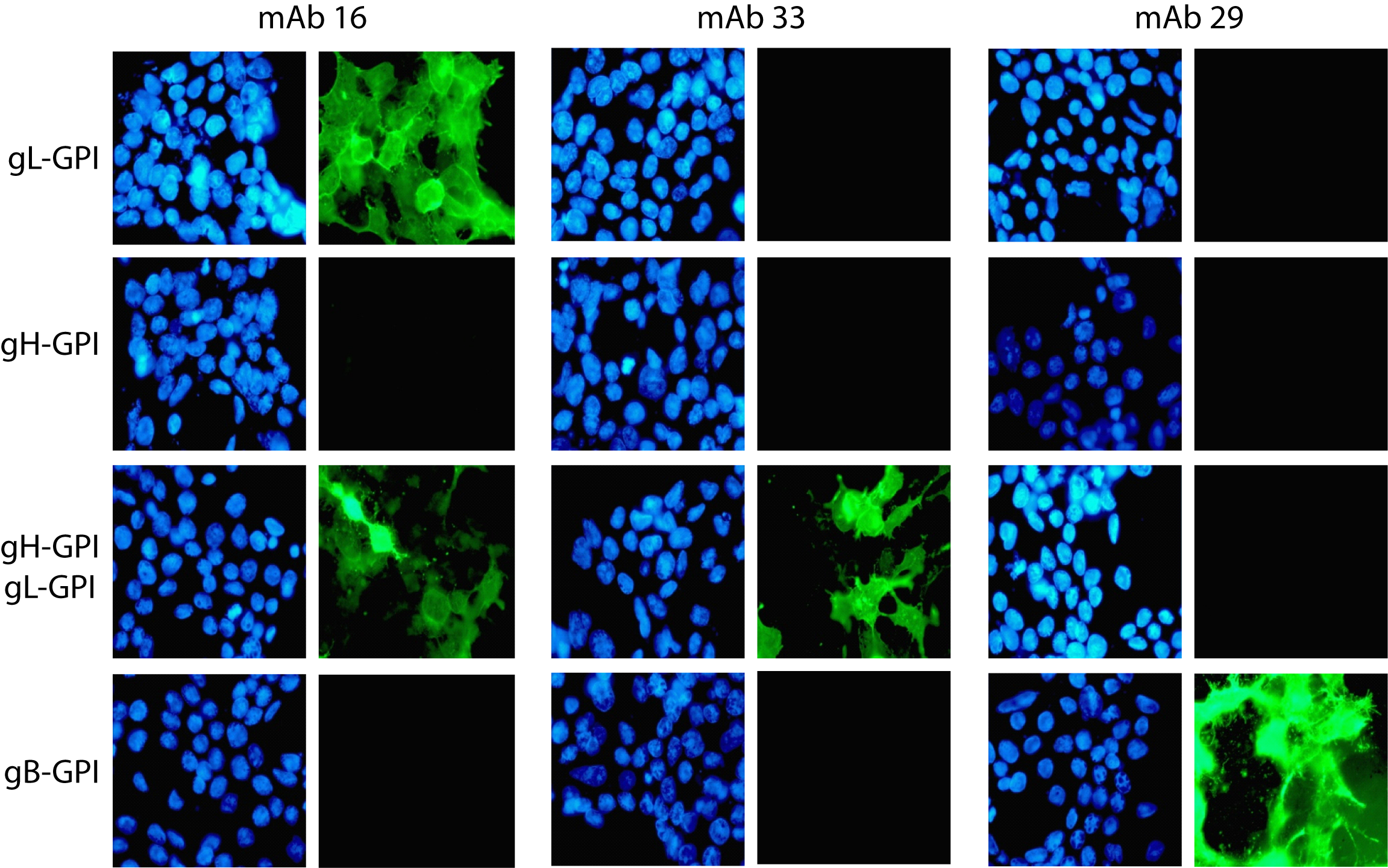

Supplement: Figure S3 — Identification of the targets of mAbs 16, 29 and 33. In order to map mAb recognition, 293T cells were transfected with the gB or gH extracellular domains or the entire gL fused to a GPI membrane anchor resulting in gB-GPI, gH-GPI and gL-GPI respectively. To reconstitute epitopes depending on the gH-gL hetrodimer, we cotransfected the cells with plasmids encoding gH-GPI and gL-GPI. Forty-eight hours after transfection, the cells were fixed and stained with the different mAbs as indicated. (TIF) [file ppat.1002387.s003.tif]

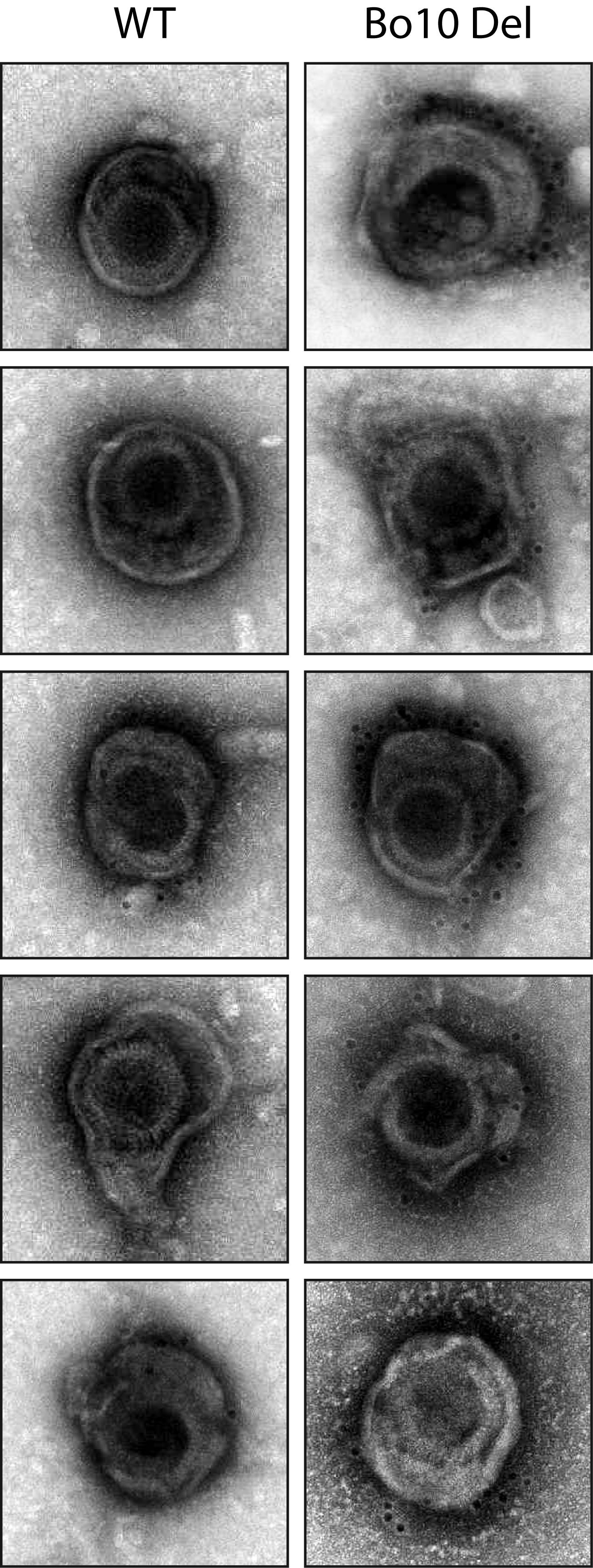

Supplement: Figure S4 — Immunoelectron microscopy of virions. Purified BoHV-4 WT V.test or Bo10 Del virions were processed for immuno-electron microscopy as described in the Material and Methods. These samples were stained for gB with mAb 35 followed by secondary goat anti-mouse IgG-10 nm gold labeled. Pictures of individual virions were then taken. Pictures of 5 representative virions per strain are showed. (TIF) [file ppat.1002387.s004.tif]

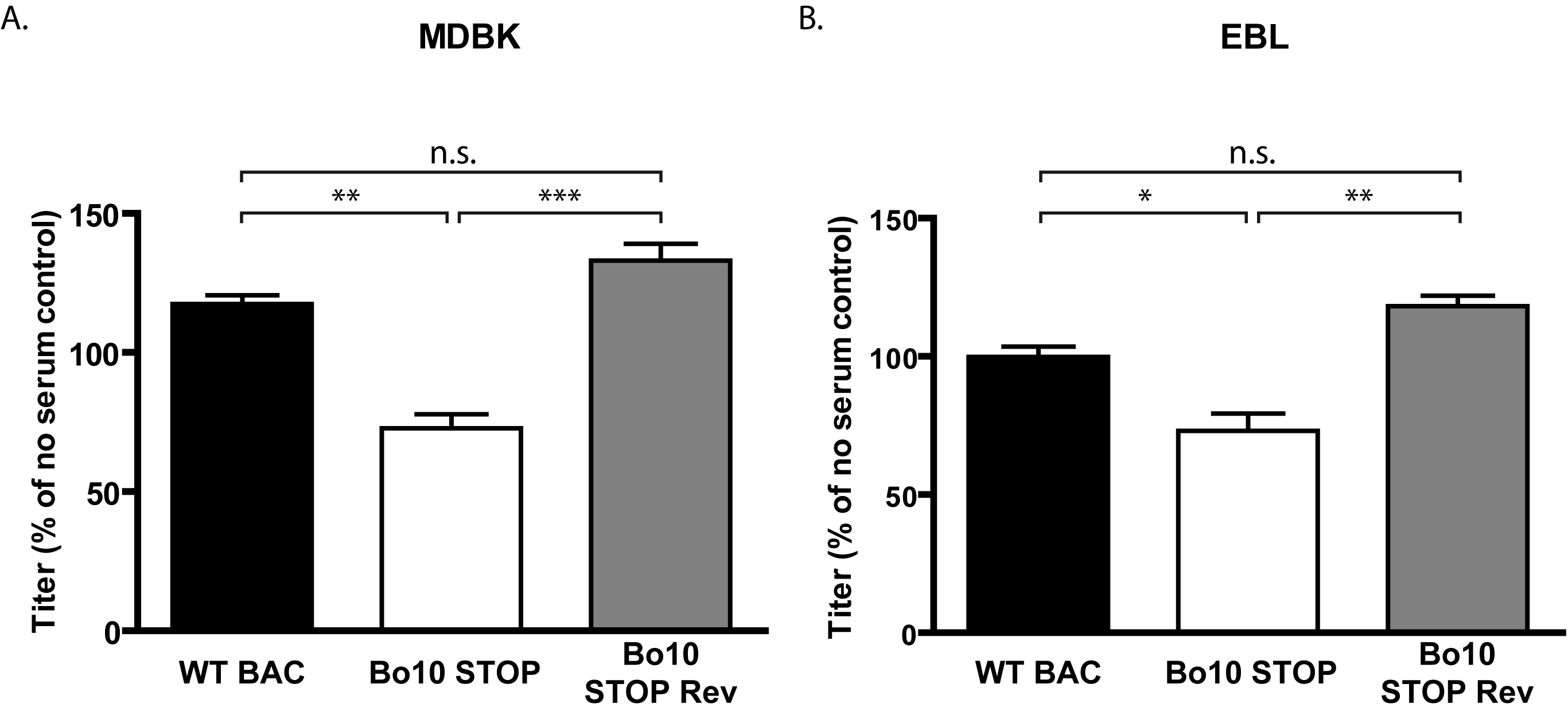

Supplement: Figure S5 — Sensitivity of the Bo10 STOP strain to anti gL directed neutralization. A. and B. BoHV-4 V.test WT BAC, Bo10 STOP and Bo10 STOP Rev virions were incubated with the gL-specific neutralizing mAb 16 (100 µg/mL). After incubation (2h, 37°C) the viruses were plaque assayed for infectivity on either MDBK cells (A) or EBL cells (B). After 4 days of incubation, the plates were fixed and the plaques were counted. BoHV-4 titers are expressed relative to virus without antibody. The data presented are the average ± SEMs for triplicate measurements and were analyzed by 1way ANOVA and Bonferroni posttests, * p<0.05, ** p<0.01, *** p<0.001. (TIF) [file ppat.1002387.s005.tif]
