# Supplementary material for: Antibody Evasion by a Gammaherpesvirus O-Glycan Shield
Source: PLoS Pathog. 2011 Nov 17;7(11):e1002387. doi: 10.1371/journal.ppat.1002387 (PMC3219721; doi:10.1371/journal.ppat.1002387)
Supplement: Table S1 — Bo10 sequences divergences among BoHV-4 strains. Nucleotide and amino acid sequences divergences of Bo10 genes from 9 BoHV-4 strains were determined using CLUSTALw. Values above and below the diagonal refer to percentages of nucleotide and amino acid sequences divergences, respectively. (DOC) [file ppat.1002387.s006.doc]

|  | V.test | LVR140 | MOVAR | 66p347 | DN599 | 108 | 130 | Buf | M40 |
| --- | --- | --- | --- | --- | --- | --- | --- | --- | --- |
| V.test |  | 0 | 0 | 1 | 1 | 14 | 14 | 11 | 11 |
| LVR140 | 0 |  | 0 | 1 | 1 | 14 | 14 | 11 | 10 |
| MOVAR | 0 | 0 |  | 1 | 1 | 14 | 14 | 11 | 10 |
| 66p347 | 1 | 1 | 1 |  | 1 | 15 | 15 | 11 | 11 |
| DN599 | 1 | 1 | 1 | 0 |  | 14 | 14 | 11 | 10 |
| 108 | 39 | 39 | 39 | 39 | 39 |  | 0 | 8 | 12 |
| 130 | 39 | 39 | 39 | 39 | 39 | 0 |  | 8 | 12 |
| Buf | 33 | 33 | 33 | 33 | 33 | 14 | 14 |  | 10 |
| M40 | 31 | 31 | 31 | 31 | 31 | 19 | 19 | 19 |  |
